# Hybrid physics-informed artificial intelligence for high-fidelity modeling and optimization of electrical systems

**DOI:** 10.3389/frai.2026.1751785

**Published:** 2026-05-28

**Authors:** Joseph Nyangon

**Affiliations:** Energy Exemplar, Salt Lake City, UT, United States

**Keywords:** electrical systems, industry 4.0, machine learning, optimization, physics-informed machine learning, physics-informed neural networks, artificial intelligence

## Abstract

The integration of machine learning with domain-specific physics has revolutionized the design, monitoring, and control of electrical machines and drives. This state-of-the-art review of physics-informed machine learning (PIML) is being leveraged to address data scarcity, improve model interpretability, and enforce physical laws, thus achieving computationally cost efficient and accurate solutions. The review also discusses challenges related to parameter sensitivity, dynamic behavior, and robustness, thereby underlining the potential of PIML to deliver computationally efficient, accurate, and scalable solutions for Industry 4.0 applications. Special emphasis is placed on hybrid physics-informed neural network (PINN) models that combine machine learning with physics-based principles for real-time diagnostics, digital twins, fault detection, and high-fidelity modeling, control and optimization of electrical machines and drives. Notable examples of these hybrid models include Deep Operator Networks (DeepONets), Fourier Neural Operators, Extreme Learning Machines (ELM)-enhanced PINNs, Graph-Based PINNs (PIGNNs), and domain decomposition PINNs, all of which illustrate the shift from traditional black-box approaches to transparent, physics-informed strategies. Through a state-of-the-art review of AI methodological contributions of PIML case studies and simulations, the potential of PIML to provide robust, scalable solutions is demonstrated, fostering a paradigm shift from black-box data-driven approaches to white-box, physics-informed strategies.

## 1 Introduction

### 1.1 Fundamentals of machine learning in electrical machines and drives

Machine learning (ML) has become increasingly important in the design, control, and optimization of electrical machines and drives, offering insights into complex processes without requiring extensive domain knowledge or prohibitive computational time (Nyangon, 2025a; Mayr et al., 2017). Contemporary applications span fault diagnostics (Kudelina et al., 2021), performance prediction (Raia et al., 2023), and control optimization (Zhang et al., 2023), with ML algorithms enabling predictive maintenance, quality management, and process control in electric drive production while supporting fast estimation of device performance during design space exploration. In power electronics specifically, ML techniques are crucial for enhancing control and optimization strategies (Bahrami and Khashroum, 2023). As the industry advances toward Industry 4.0 standards, the integration of ML into electrical machines and drives is expected to become more prevalent, but its full potential is realized only when paired with physical modeling—a synergy that motivates the shift toward hybrid physics-informed artificial intelligence for high-fidelity simulation and optimization. Within this trajectory, artificial neural networks (ANNs) have driven progress in complex system identification, control, and estimation, finding applications in static feedback signal estimation, space vector PWM, and flux vector estimation (Bose, 2020). Convolutional Neural Networks (CNNs) have shown particular promise for diagnosing bearing faults and detecting winding defects in electric motors (Husebø et al., 2019), classification in manufactured parts (Warren et al., 2021), and pattern recognition in power electronic systems (Abu-Rub et al., 2021), with CNN–batch normalization integration further improving fault detection in induction motors (Kumar and Shankar Hati, 2021). Despite challenges around data quality and real-time implementation (Khashroum et al., 2023), these neural approaches offer substantial potential for reliable, automated fault diagnosis – an outcome amplified when underlying physics is embedded into the learning framework.

Before the deep learning era, ML applications in electric machine drives relied on hardware platforms optimized for high-speed, parallel processing, although many implementations were constrained by slower, sequentially operated digital signal processors (Mojlish et al., 2017). Embedded platforms such as field-programmable gate arrays (FPGAs), graphics processing units (GPUs), and chip multiprocessors—now central to real-time simulation and control of electric machines—were not yet fully developed and thus saw limited use, despite their advantages in simulation acceleration and computational efficiency (Osta et al., 2019). Traditional ML techniques such as support vector machines, neural networks, and random forests were applied to predictive maintenance and quality management (Mayr et al., 2017), but the advent of deep learning has driven a shift toward more advanced algorithms and specialized hardware, enabling improved control and monitoring (Zhang et al., 2023) and an increased focus on hardware-aware ML modeling and optimization (Marculescu et al., 2018). Hardware bottlenecks nonetheless remain a significant barrier in industrial settings, often producing suboptimal performance in identification and control tasks. Recent advances in FPGAs are mitigating these constraints by offering substantial gains in throughput, latency, and energy efficiency relative to CPUs and GPUs (Kathail, 2020), with high parallelism, low latency, and hardware customization making them well suited to accelerating ML algorithms in autonomous driving, healthcare, and electronic design automation (Itagi et al., 2021). Their integration is improving control and monitoring of electric machine drives (Zhang et al., 2023) while supporting emerging applications such as power-electronics-dominated grids (Nyangon, 2025b; Abu-Rub et al., 2021), data-driven thermal modeling of power electronic modules (Li et al., 2023), and anomaly detection (Hossein Rahimighazvini et al., 2024).

As embedded systems mature, data-driven approaches are becoming increasingly attractive for high-performance control, and model-compression techniques are essential for translating complex neural networks into deployable solutions. Pruning and quantization can significantly reduce model size and improve inference speed without substantial accuracy loss by eliminating low-impact parameters and reducing numerical precision (Loureiro et al., 2023). Quantization-aware pruning has achieved size reductions of up to 94% with 73% faster inference (Torres-Tello and Ko, 2022), while deep compression—integrating pruning, quantization, and Huffman coding—has reduced model sizes by 35 × to 49 × without performance loss (Han et al., 2015), enabling deployment on resource-constrained edge devices (Loureiro et al., 2023). Scientific ML, parallelization, and high-performance computing further accelerate these workflows for large-scale networks. Crucially, physics-informed learning enhances these capabilities by enabling online estimation of unknown or time-varying system parameters and rapidly solving for system states during transient dynamics and stability analysis—uniting compact, hardware-efficient models with rigorous physical consistency to advance high-fidelity modeling and optimization of electrical machines.

### 1.2 Physics-informed machine learning

PIML integrates physical laws and domain knowledge into ML models, enhancing their accuracy, efficiency, and optimizability (Nyangon, 2025a; Nghiem et al., 2023; Pateras et al., 2023). PIML offers benefits such as improved data efficiency, physical consistency, and interpretability (Meng et al., 2022). It has been successfully applied in various fields, including weather and climate modeling (Nyangon, 2024), tribology (Marian and Tremmel, 2023), and fluid mechanics (Sharma et al., 2023). PIML approaches can be broadly categorized based on how physics information is derived and incorporated into the learning process (Pateras et al., 2023). Common implementations include physics-informed neural networks and physics-informed graph networks (Shukla et al., 2022). While PIML shows promise in addressing complex multiscale problems, challenges remain in developing robust and reliable models for large-scale engineering applications (Asef and Vagg, 2024).

#### 1.2.1 Physics-informed neural networks

**Figure 1:**
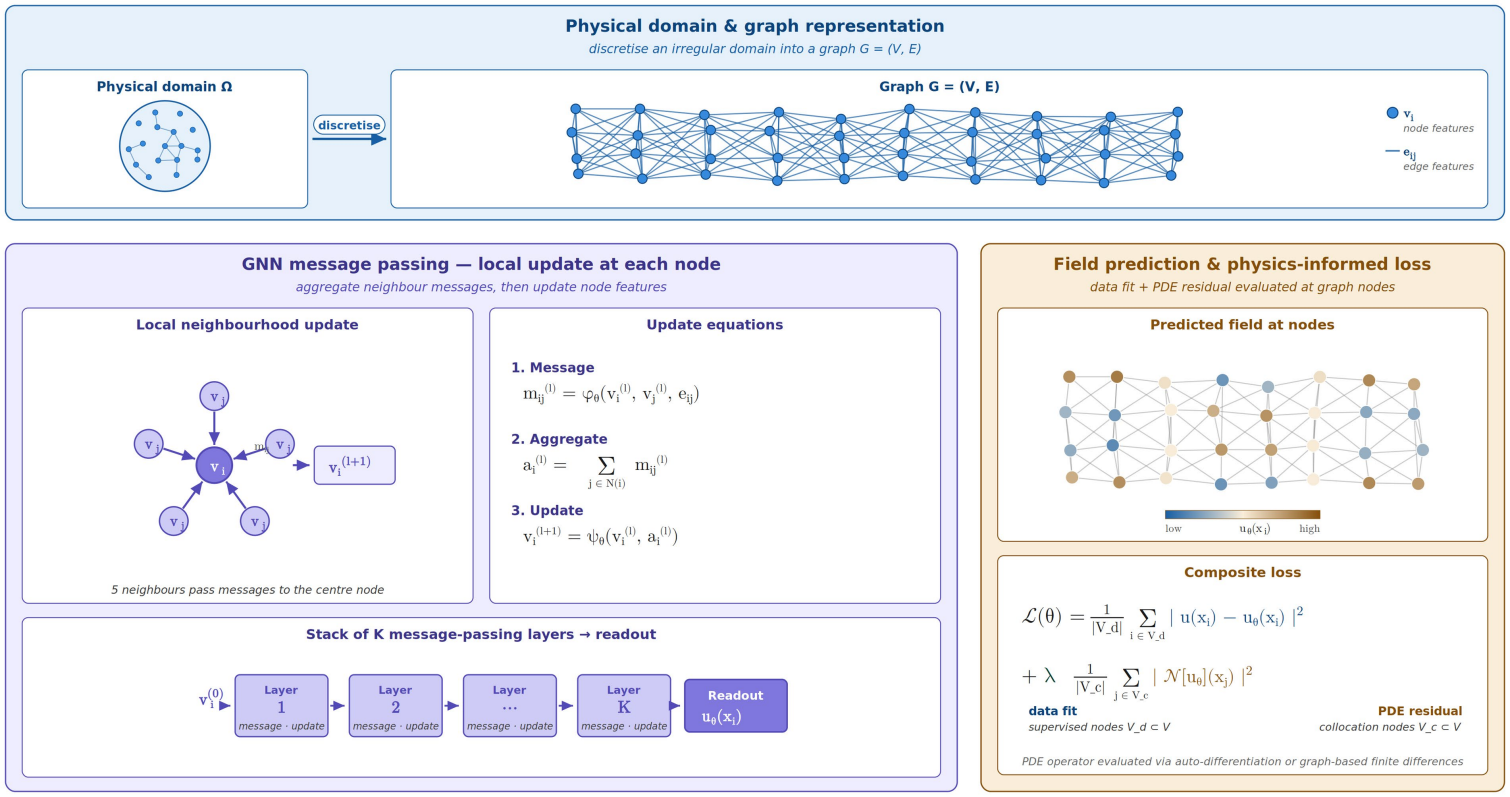
PINNs loss formation.

PINNs are a deep learning framework that integrates physical laws described by parametrized partial differential equations (PDEs) into neural network architectures by incorporating the residuals of the underlying equations as additional loss terms during training, enabling networks to either encode physical laws or infer them from data (Nyangon, 2025a; Raissi et al., 2019). A common formulation of PINNs combines a data-fidelity loss with a physics loss governed by a differential operator and balanced through a weighting hyperparameter (Figure 1). This dual objective encourages the model to fit observed data while adhering to the governing physics. Although PINNs have shown promise as function approximators, their application to highly nonlinear, chaotic, or multiscale problems has exposed challenges in stability, convergence, and gradient pathologies (Wang et al., 2021). Recent work has addressed these issues through specialized activation functions, advanced optimization techniques, and refined loss structures (Antonion et al., 2024; Cuomo et al., 2022), enabling PINNs to model complex systems such as the Lorenz system, the Kuramoto–Sivashinsky equation, and the Navier–Stokes equations governing turbulent flow. Nevertheless, these solutions often address isolated challenges rather than ensuring broad applicability, leaving generalizability and convergence as active research frontiers.

#### 1.2.2 Physics-informed graph neural networks

**Figure 2:**
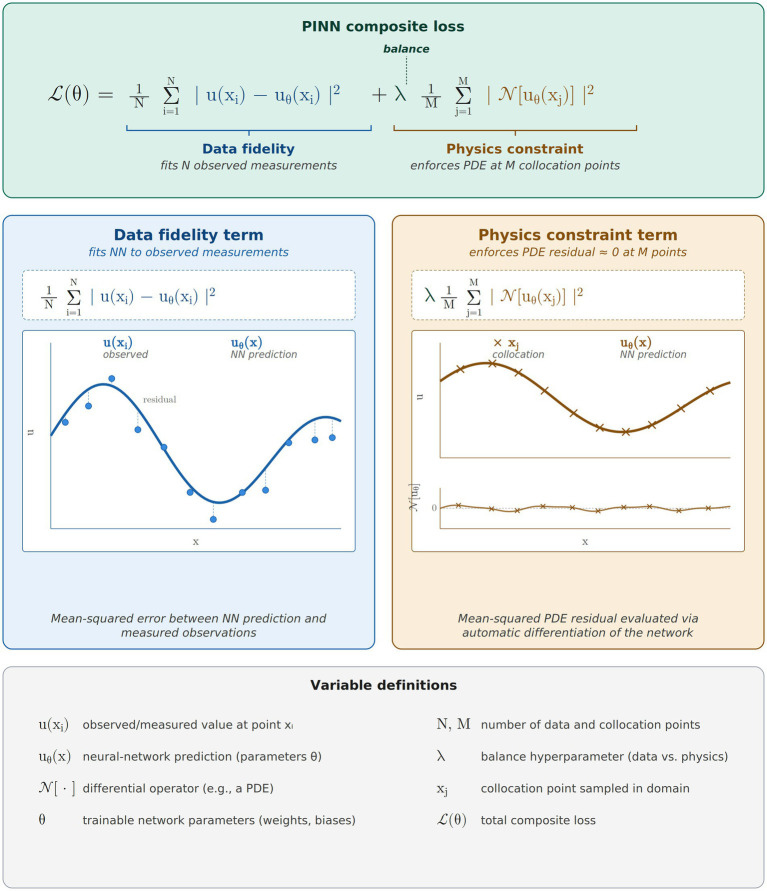
Graphical illustration of PIGNNs interdependencies (author’s illustration).

PIGNNs have emerged as a robust framework for addressing complex dynamical systems by combining the intrinsic advantages of graph neural networks with physics-based constraints (Nyangon, 2025a). The architecture integrates prior knowledge of physical laws to enforce consistency in predictions, ensuring that outputs remain aligned with fundamental governing principles, and is particularly effective at handling irregular meshes, accommodating longer time steps, and adapting to varying initial and boundary conditions. These attributes yield improved accuracy and enhanced data efficiency when simulating system dynamics, including the preservation of conserved quantities. Recent innovations—such as incorporating causality during training and integrating Bayesian inference for uncertainty quantification (Linka et al., 2022) – have expanded PIGNN applicability across structural dynamics, control problems, and nonlinear system analysis (Antonelo et al., 2024), improving performance in both forward and inverse problems while offering a promising pathway toward zero-shot scalability to larger system sizes. Architecturally, PIGNNs leverage graph structures—nodes and edges—to represent spatial relationships and connectivity, aggregating local information through convolutional and attention-based mechanisms (Figure 2; Sen et al., 2024). The adjacency matrix, either informed by domain expertise or self-learned, plays a critical role in quantifying relationships among system components. The framework is designed to address data limitations such as missing or incomplete inputs, although accuracy deteriorates when influential variables (e.g., inlet vessel information in fluid dynamics) are absent, underscoring the necessity of precise data integration (Wang et al., 2021). By embedding physics constraints directly into training, PIGNNs mitigate extrapolation risks and enhance interpretability, balancing computational efficiency with physical fidelity (Cuomo et al., 2022; Shukla et al., 2022).

### 1.3 Scope and outline of the study

The motivation for applying PIML in the design and control of electrical machines and drives is driven by the need to reconcile data-driven approaches with the immutable laws of physics. By integrating physical laws with ML models, this approach addresses data scarcity and model generalizability while ensuring physical plausibility (Meng et al., 2022), and advanced methods such as domain decomposition and neural operator learning further demonstrate PIML’s capacity to tackle high-dimensional problems that traditional numerical methods often find intractable (Pateras et al., 2023). These capabilities enable rapid optimization of complex geometries and control strategies, improving torque, efficiency, and overall system reliability. This paper presents a state-of-the-art review and categorization of recent PIML advances for electrical machines and drives, organized around three themes: the AI methodological contributions of PIML, the motivation of integrating physical knowledge into ML frameworks, and the methodological approaches employed. Current challenges—including data scarcity, optimizability, and the computational expense of deep learning models (Hao et al., 2023)–are discussed alongside opportunities for real-time deployment in embedded systems, with applications spanning system identification, control optimization, and digital twins (Nghiem et al., 2023). The remainder of the paper is structured as follows: Section 1 introduces the fundamentals of PIML for electrical machines and drives alongside key industrial machines and their challenges; Sections 2 and 3 present the state-of-the-art review of PIML techniques; Section 4 discusses challenges and future trends; and Section 5 concludes.

## 2 Fundamentals of PIML for electrical machines and drives

### 2.1 Overview of electrical machines and drives

Electrical machines and drives are essential components in energy conversion systems, transforming electrical energy into mechanical energy and vice versa (De Doncker et al., 2011). At their core, these systems encompass a range of devices—from AC, DC, synchronous, to induction machines—each tailored for specific applications through unique operational characteristics and performance benefits. Fundamental terminologies such as power, torque, speed, efficiency, and control, underpin the understanding of these devices, where concepts like Faraday’s law and the Lorentz force elucidate the conversion processes between electrical and mechanical energy. In motors, electromagnetic interactions produce rotational motion, whereas in generators the same principles facilitate the conversion of mechanical motion into electrical output. Modern drive technologies, including variable frequency drives, vector control, and direct torque control, further refine the performance and efficiency of these systems by enabling precise regulation of speed, torque, and direction (Chen and Tong, 2023). This intricate interplay between classification, operating principles, and control strategies underlines the technological advancements that continue to drive improvements in performance and application versatility.

**Figure 3:**
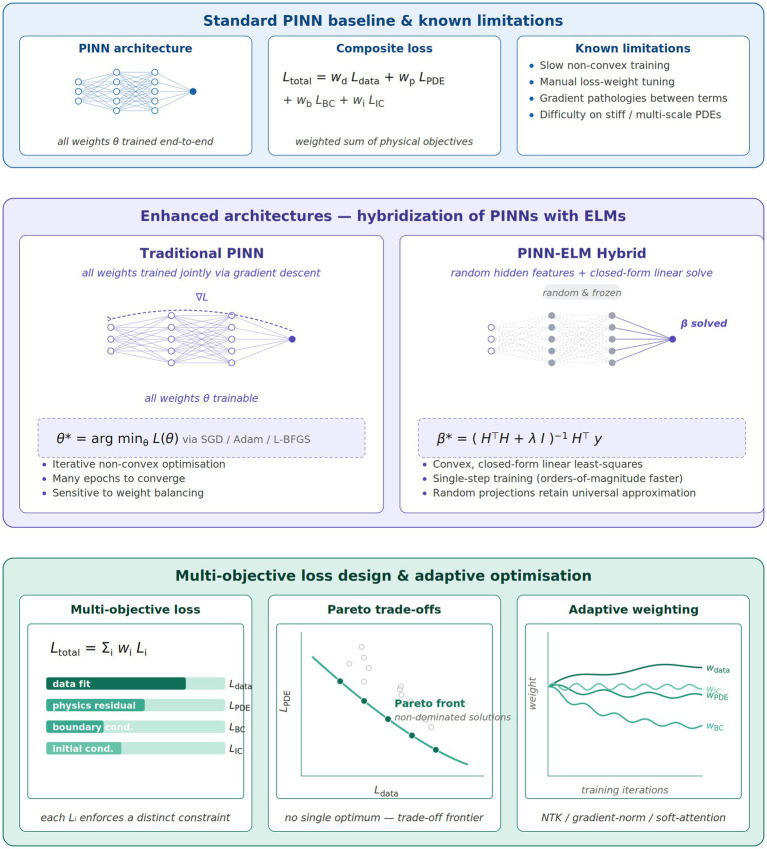
Categorization of electrical machines and drives by application and performance characteristics.

Design considerations for electrical machines and drives focus on key parameters such as efficiency, power density, and dynamic response across four areas (Figure 3): (a) industrial and general-purpose applications, (b) high-performance and precision control applications, (c) energy-efficient and modern motor technologies, and (d) specialized and harsh environment applications, aligning machine capabilities with specific application demands (Boldea et al., 2018). These design imperatives are evident in a wide range of applications—from electric vehicles and industrial automation to renewable energy systems—where integration with modern control systems and smart grid technologies is increasingly prevalent (Nyangon and Darekar, 2024). For instance, industrial settings often favor robust induction motors for their durability, while permanent magnet motors are preferred in electric vehicles for their improved efficiency and power density. Simultaneously, emerging trends highlight advancements in materials, digital control, and energy-efficient technologies, suggesting a future where even higher performance and adaptability become standard. Innovations in design and the evolving role of drive systems are poised to further enhance operational reliability and sustainability, ensuring that electrical machines continue to meet the growing demands of modern energy conversion challenges.

#### 2.1.1 Industrial and general-purpose applications

AC induction motors are widely employed across industrial and general purpose applications due to their simple construction, inherent robustness, and favorable cost–performance trade-offs across domestic, automotive, petrochemical, and oil-and-gas settings (Chen and Tong, 2023; Faiz et al., 2017). Three-phase stator windings coupled to squirrel-cage rotors enable efficient energy conversion under wide load variability, while closed-loop control ensures accurate torque, speed, and position regulation with reversible motor–generator operation—critical for resilience and lifecycle economics relative to brushed alternatives (Boldea et al., 2018). Recent. Field-weakening extends speed beyond base values and supports scalability from fractional-kilowatt appliances to multi-megawatt compressors, whereas variable-frequency operation suppresses inrush, stabilizes thermal profiles, and improves load-matching efficiency in continuous and cyclic duty. Reliability in hazardous atmospheres mandates explosion-proof or pressurized enclosures to mitigate ignition risks in Class I/Division 1 or Zone 1 applications; compliance engineering adds cost and weight but preserves availability in mission-critical service (Mistry et al., 2024). Ongoing research targets higher power density, reduced losses, and advanced observers for parameter drift, thereby strengthening condition-based maintenance and extending overhaul intervals without eroding capital efficiency (Chen and Tong, 2023). Collectively, these attributes—ruggedness, scalability, and controllability—explain the enduring centrality of induction machines in electrified systems spanning pumps, fans, conveyors, and subsea lift where mean-time-between-failure and total cost of ownership dominate specification.

Variable-frequency drives (VFDs) and soft starters provide complementary actuation layers that tune electrical and mechanical stress to duty profiles, improving efficiency, reliability, and asset life at system level. VFDs modulate voltage and frequency to align electromagnetic torque with instantaneous demand, enabling soft-start, load-shedding, and speed optimization in upstream oil and gas (beam pumping, FPSOs), petrochemicals, hydrogen process auxiliaries, HVAC, and transport—where energy savings and harmonics compliance must be balanced against over-torque and partial-load losses (Nyangon and Darekar, 2024; Tan et al., 2020). Recent work refines control algorithms and protection logics to harden drives against grid disturbances and non-ideal waveforms, lifting availability in remote or islanded power systems (Guo et al., 2024). Soft starters, by contrast, regulate input voltage at fixed frequency using thyristor stages to curtail starting current, mitigate torque ripple, and improve input power factor with minimal component count and attractive first cost (Nannen et al., 2022). They have scaled across fans, pumps, compressors, mixers, and electric submersible pumps in aerospace, manufacturing, and oil and gas, while research advances—three-phase PWM AC choppers—reduce sensors and harmonic distortion to limit rotor heating and bearing stress (Öksüztepe et al., 2022). In combination, VFDs deliver maximal energy optimization and flexibility; soft starters supply cost-effective, reliable starts – together furnishing robust, scalable drive solutions across duty classes.

#### 2.1.2 High-performance and precision control applications

Permanent-magnet synchronous machines (PMSM) and their associated vector-controlled drives underpin high-performance motion systems in electric vehicles, industrial automation, CNC tooling, and robotics, where strict torque and speed accuracy, low latency, and compact integration are paramount (Ali et al., 2024). A laminated stator carrying three-phase windings establishes a rotating field that locks synchronously with rotor-mounted permanent magnets, yielding high power density, excellent efficiency, a wide constant-torque range, and inherently smooth torque production essential for contouring and pick-and-place tasks. In this regime, field-oriented control decouples flux and torque channels, enabling linearized current-to-torque behavior and fast dynamic response under load disturbances, while modern estimators preserve precision at partial load and low speed (Zhu et al., 2021). The absence of brushes reduces maintenance and acoustic noise, and the compact electromagnetic package promotes installation in space-constrained end-effectors and aerospace actuators. Recent work integrates machine-learning-assisted parameter identification and on-line thermal models to stabilize bandwidth despite magnet temperature drift and inverter non-idealities, improving transient and steady-state error budgets. These attributes translate directly into higher throughput and tighter tolerance in precision assembly cells, wafer handling, and coordinated multi-axis gantries. Consequently, PMSM platforms, paired with vector control, form the benchmark for energy-efficient servo axes, delivering repeatable micrometer-class positioning and millisecond-scale torque steps while containing switching losses through optimal modulation and advanced observers.

Direct-torque-controlled drives complement vector-controlled PMSM systems where near-instantaneous torque steps, minimal parameter dependence, and robust disturbance rejection are required, as in high-speed pick-and-place, robotic joints, and spindle drives for CNC machining (Meesala and Thippiripati, 2020). By regulating electromagnetic torque and stator flux directly, DTC avoids coordinate transformations and current-loop cascades, achieving superior transient behavior at the expense of characteristic torque ripple and variable switching frequency; contemporary schemes moderate these effects using constant-frequency modulators, refined look-up tables, and virtual voltage space vectors (Hakami et al., 2019). Dual-inverter and multi-level variants suppress acoustic noise and improve efficiency under wide speed ranges and fluctuating loads, consolidating DTC’s role in electric vehicles, high-power induction drives, and industrial actuators. On the application layer, servo drives integrate these control primitives—field-oriented, DTC, and model predictive control—behind unified motion controllers providing precise speed, acceleration, and position profiles with jerk constraints, feed-forward shaping, and synchronized multi-axis interpolation (Kitayoshi et al., 2023). Coupled with industrial networks and IoT gateways, modern servo platforms stream telemetry for condition monitoring, on-line identification, and predictive maintenance, thereby sustaining bandwidth and positioning accuracy as mechanisms wear and thermal states drift. Emerging direct-drive architectures and multi-degree-of-freedom stages extend stiffness and back-drivability benefits to collaborative robots and precision metrology while reducing backlash, gear noise, and maintenance. Together, these advances deliver repeatable sub-arcsecond positioning, low settling times, and energy-aware motion, enabling higher utilization, safer human-robot collaboration, and consistent quality across automation cells.

#### 2.1.3 Energy-efficient and modern motor technologies

Synchronous Reluctance Motors (SynRMs) are AC machines without rotor windings or magnets, yielding compact, low-loss designs with high torque density and lower cost (Bianchi et al., 2021). Recent optimization of rotor saliency and flux-barrier geometry mitigates torque ripple and improves power factor, closing the gap between theoretical and realized performance (Murataliyev et al., 2022). In industrial duty—fans, pumps, mills—SynRMs increasingly outperform induction motors on efficiency, enabling smaller frames, reduced cooling demand, and lower lifetime energy spend. Their magnet-free bill of materials reduces rare-earth risk and aligns with green-tech procurement goals. When paired with modern vector drives, SynRMs deliver accurate speed control and strong low-speed torque, suiting EV auxiliaries, HVAC, and process control. Ongoing materials and control advances position SynRMs as an emergent topology across energy-intensive sectors (Bianchi et al., 2021).

Brushless DC (BLDC) motors are permanent-magnet machines that eliminate brushes and commutators, reducing copper loss, friction and maintenance while improving efficiency and power density. Contemporary drive strategies—field-oriented control, direct-torque control and model-predictive control—suppress torque ripple, enhance low-speed smoothness, and minimize switching loss to optimize energy use (Mohanraj et al., 2022). The result is compact actuators with high torque-to-weight ratios for EV traction and auxiliaries, UAVs, robotics, haptics, pumps, and HVAC compressors, where partial-load efficiency is critical. Advances in semiconductor packaging, current sensing and calibration further improve dynamic response and EMI robustness, widening suitability for HVAC, manufacturing and green-tech platforms. Remaining challenges—thermal bottlenecks in dense stators and fault diagnostics in sensor-limited systems—are active areas of research, with promising results in estimation-based protection and enhanced cooling (Shokri and Naderi, 2017). As costs fall and controls mature, BLDC drives will continue to displace induction solutions in efficiency-critical applications.

#### 2.1.4 Specialized and harsh environment applications

Switched reluctance motors (SRMs) exploit variable reluctance and a salient-pole rotor, yielding a simple, magnet-free topology tolerant to heat, vibration and contamination. Their robust laminations, mechanical clearances and thermally resilient windings support sustained operation under high-temperature duty cycles and shock-prone settings typical of EV powertrains, aerospace actuators, wind generator pitch drives and heavy-duty appliances (Li et al., 2019). Absence of rare-earth magnets eliminates demagnetization risk at elevated temperatures and reduces cost volatility, while improving thermal headroom for higher current densities. Modern control—especially model predictive control—and multiphase configurations suppress torque ripple and acoustic noise, enhance fault tolerance, and stabilize nonlinear behavior across wide speed ranges (Valencia et al., 2021). Collectively, these attributes position SRMs as reliable, efficient alternatives to induction and permanent-magnet machines for harsh-environment duty, with manufacturability and lifecycle economics (Diao et al., 2022). Table 1 summarizes major electrical machines, drives, applications and advantages.

**Table 1:** Electrical machines, drives, applications and PIML models.

Component	Applications	Advantages	Representatives
AC induction motors	Industrial machinery, HVAC systems, pumps, fans	Robust, cost-effective, high reliability	PINNs for system identification
PMSM	Robotics, electric vehicles, precision instrumentation	High efficiency, improved performance, precise control	Hybrid models combining physics-based simulation with deep learning
SynRMs	Energy-efficient industrial applications, fans, pumps	Improved efficiency, reduced maintenance costs	PINN-enhanced optimization models
SRMs	Harsh environment applications, automotive, industrial systems	Simple design, robust under extreme conditions	Data-driven approaches with embedded physical constraints (PINNs)
BLDC	Household appliances, robotics, industrial automation, medical devices	High reliability, precise control, energy-efficient	Physics-informed hybrid deep learning models
VFD	AC motor control in industrial, HVAC, and pump applications	Energy efficiency, variable speed control, and reduced power consumption	PINN-based predictive maintenance and control models
Vector control drives	Robotics, precision manufacturing, high-performance industrial systems	Enhanced torque and speed regulation, improved dynamic response	Hybrid PINN models for advanced control strategies
DTC drives	High dynamic industrial applications, traction control systems	Rapid torque response, improved dynamic performance	Real-time PIML integration for torque and efficiency optimization
Servo drives	Robotics, CNC machinery, automation systems	High precision, fast dynamic response, accurate motion control	Digital twins with embedded PIML for predictive maintenance and control
Soft starters	Motor startup applications in industrial and commercial settings	Controlled acceleration, reduced mechanical stress and inrush currents	PINN-based anomaly detection, predictive control models and early fault detection

### 2.2 PIML and operator learning for electromagnetic field analysis of electrical machines

**Figure 4:**
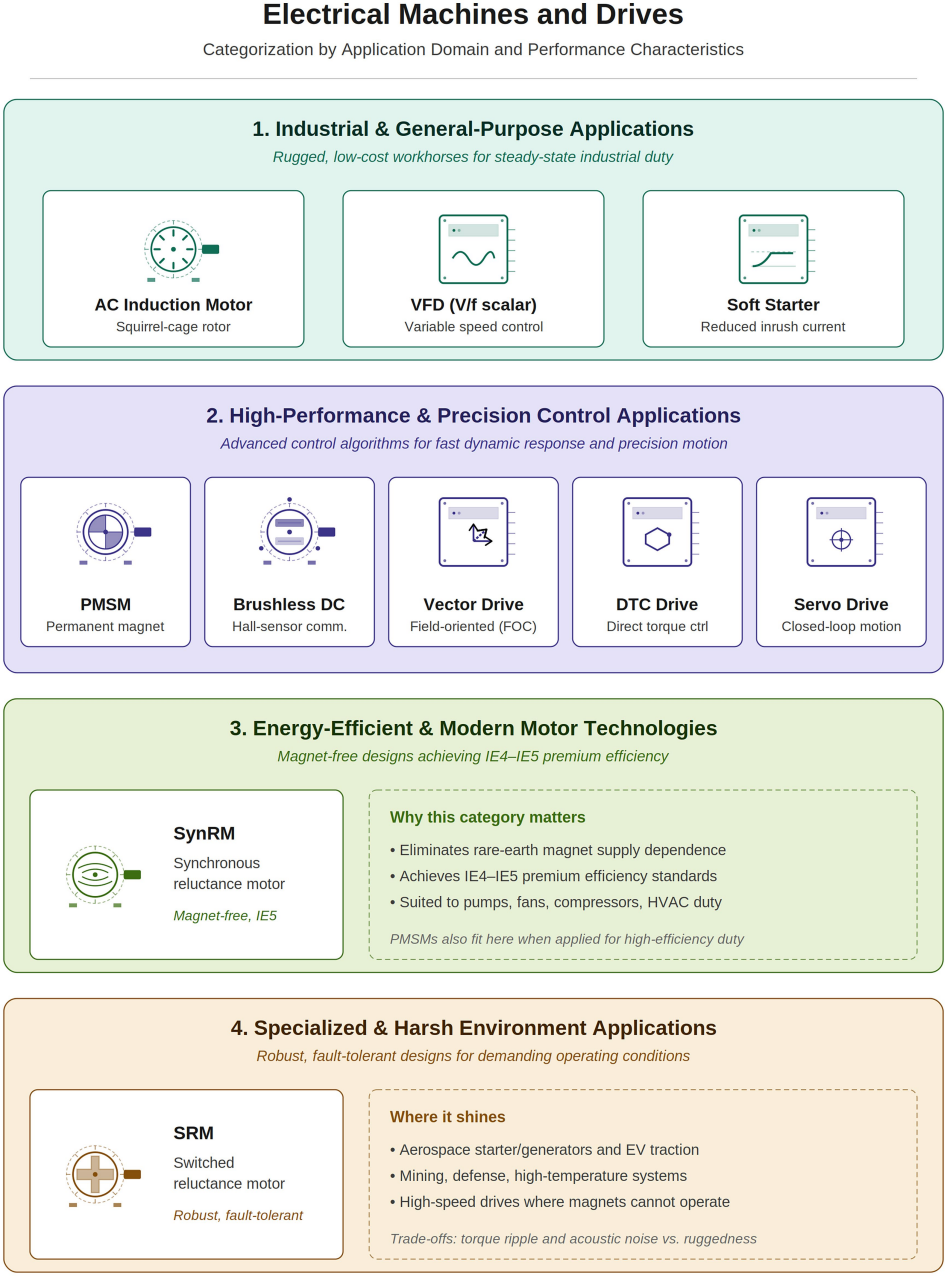
PIML and operator learning for electromagnetic analysis of electrical machines.

Recent advancements in PIML have significantly enhanced its application across diverse scientific fields, including tribology, fluid mechanics, and chemical engineering (Sharma et al., 2023). By embedding physical laws into ML frameworks, PIML offers improved accuracy, interpretability, and transferability over traditional data-driven methods (Nyangon, 2025a; Karniadakis et al., 2021). Figure 4 illustrates a graphic linking PIML and operator learning to outputs, showing physics/data losses, operator nets, hybrid surrogate, and active-learning feedback for electromagnetic machine analysis and deployment. Key advances include the evolution of PINNs (section 1.2.1) and PIGNNs (section 1.2.2), which enable the resolution of complex multi-physics problems and the discovery of hidden physics (Shukla et al., 2022). Further progress has been made with: (i) enhanced PINN architectures with methods such as Extreme Learning Machines (ELM), (ii) neural operator techniques for learning mappings between function spaces beyond solving partials pointwise, (iii) novel approaches in domain decomposition and modular frameworks, (iv) adaptive optimization, multi-fidelity strategies, and (v) the integration of functional interpolation techniques with PINN for increased data efficiency and stabilized predictions (see Supplementary Sections 1.1, 1.2). These improvements have been demonstrated in seismic wave modeling, multi-physics coupling in chemical engineering, and anomaly detection in smart grids (Zideh et al., 2024; Nyangon and Akintunde, 2024). Table 2 details recent advancements in PIML along with their technical innovations, benefits, and the areas of application.

**Table 2:** Recent advances in PIML methods: techniques, benefits, and applications.

Recent advancement	Key features and recent developments	Impact and benefits	Applications
Evolution of PINNs	Adaptive weighting and multi-scale feature extraction. Enhanced loss functions, automatic differentiation.	Improved PDE convergence Robustness in diverse applications	Fluid dynamics Heat transfer Structural mechanics
Advances in PIGNNs	Graph-based spatial interaction modeling. Multi-physics interaction modeling to uncover hidden physics.	Solves complex multi-physics problems Discovers latent physical laws	Electrical networks Biological networks Material science
Enhanced PINN architectures with ELM	Integrates ELMs to expedite training with randomly weighted hidden layers Analytic output weighting.	Reduces training time, lower computation costs Improved generalization.	Real-time simulations Process control Engineering prototyping
Neural operator techniques	Learns mapping between function spaces DeepONet and Fourier Neural Operators (FNO) that capture global solution behaviors.	Efficient high-dimensional problem solving Captures global solution behaviors	Weather prediction Climate modeling Uncertainty quantification
Domain decomposition and modular frameworks	Subdomains solved by modular PINNs. Parallel training for heterogenous systems.	Scalable for large problems Better boundary or interface handling	Structural analysis Geophysical simulations Industrial processes
Adaptive optimization and multi-fidelity strategies	Adaptive learning rate and sampling. Multi-fidelity data integration	Efficient training and accurate models. Cost-effective simulations	Aerospace design Automotive engineering Environmental modeling
Functional interpolation with PINNs (see Supplementary Section 1.5).	Couples PINNs with interpolation methods (e.g., kernel-based techniques) to enhance performance on sparse or noisy datasets. Stabilizes predictions with functional priors	Increased data efficiency and stability of predictions Reliable results in under-sampled or noisy data environments.	Biomedical imaging Geostatistics Remote sensing

Figure 4 illustrates a workflow linking PIML and operator learning (see Supplementary Section 1.3) to predict electromagnetic fields, drive optimization and real-time deployment, featuring active-learning feedback from outputs.

### 2.3 The benefits of PIML in engineering applications

By incorporating domain-specific knowledge directly into the learning process, PIML significantly enhances data efficiency, thereby reducing the reliance on vast datasets while maintaining high fidelity in modeling complex phenomena (Wu et al., 2024). This integration not only improves the interpretability of the models but also ensures that predictions remain consistent with known physical principles, which is crucial for applications ranging from fluid mechanics to structural integrity (Marian and Tremmel, 2023; Sharma et al., 2023). Furthermore, the ability of PIML techniques—such as PINNs and PIGNNs—to implicitly capture underlying dynamics presents a promising avenue for discovering hidden physics in systems with sparse or noisy data, thus offering a robust framework for addressing multi-physics challenges.

In addition, PIML provides substantial benefits by streamlining the simulation of complex engineering processes through a reduction in computational overhead and enhanced generalizability. The methodology allows for the seamless integration of multimodality and multi-fidelity data, ensuring that even limited datasets yield reliable and physically consistent predictions (Wu et al., 2024). This capacity to incorporate prior physical knowledge reduces the need for exhaustive data re-learning, thereby expediting the modeling of transport processes, chemical reactions, and structural behavior (Shukla et al., 2022). By doing so, PIML not only advances the precision of predictive models but also fosters innovative solutions for design, maintenance, and asset management in engineering, marking a significant leap forward in the field (Zhao et al., 2020).

Table 3 summarizes the benefits of PIML in engineering applications, demonstrating a paradigm shift through the integration of physical laws such as computational fluid dynamics (CFD) and thermal systems, enhanced data efficiency, and improved interpretability and trustworthiness. By embedding governing physical principles into model architectures, loss functions and optimization routines, PIML not only reduces the solution space but also stabilizes predictions in scenarios with scarce or noisy data (Sharma et al., 2023). This approach facilitates superior generalization and extrapolation capabilities, enabling accurate inference in complex, high-dimensional multi-physics problems (Nyangon, 2025a; Karniadakis et al., 2021). Furthermore, rigorous uncertainty quantification is achieved by leveraging physical constraints, thereby bolstering model trustworthiness and reliability (Mao and Jin, 2024). In addition, PIML offers computational efficiency by diminishing the need for extensive datasets and accelerating convergence, while ensuring robust model stability. Finally, comprehensive validation and verification protocols ensure that these models maintain both transparency and interpretability, fostering increased confidence in their deployment for diverse engineering challenges (Xie et al., 2023), thus promoting innovation.

**Table 3:** The benefits of modern PIML in engineering: enhancing accuracy, efficiency, and predictive capabilities.

Benefits of PIML	Key considerations	Engineering applications	PIML Model	References
Integration of physical laws	Embed physics; use hybrid modeling	CFD, structural mechanics, thermal systems	PINNs: Navier–Stokes solver	Gu et al. (2024)
Data efficiency and scarcity	Reduce data; balance quality and quantity	Structural monitoring, aerospace design	PINNs predict stress from sparse sensors	Cheruku et al. (2025) and Mao and Jin (2024); Sharma et al. (2023)
Interpretability and trustworthiness	Ensure physics consistency; boost explainability	Predictive maintenance, robotics, process control	Hybrid model for interpretable diagnostics	Ali et al. (2024) and Wu et al. (2024)
Generalization and extrapolation	Robust across regimes; transferable modeling	Multi-physics, environmental, energy systems	Optimizable PINNs for varying boundaries	Nyangon (2025a) and Torchio et al. (2025)
Uncertainty quantification	Propagate errors; integrate probabilistic measures	Risk assessment, safety-critical simulations	Bayesian PINNs provide uncertainty bounds	Huber et al. (2023) and Ma et al. (2022)
Computational efficiency	Balance physics solvers and data efficiency	Real-time monitoring, control engineering	Reduced-order PINNs enable fast real-time simulation	Cheruku et al. (2025)
Model stability and convergence	Ensure numerical stability; optimize convergence	Stability analysis, vibration studies, circuit simulations	Adaptive PINNs improve training convergence	Gu et al. (2024) and Yin et al. (2024)
Validation and verification	Benchmark rigorously; perform systematic error analysis	Calibration in automotive, aerospace, energy systems	PIML cross-validated against experimental data	Nyangon (2025a) and Diz et al. (2023)

## 3 Applications of PIML in electrical machines and drives

### 3.1 Field analysis: theory, data, and robustness

Electromagnetic field simulations are finding ubiquitous applications in the design and analysis of electrical machines and drives. Their ability to predict torque, flux, and force distributions across complex geometries makes them indispensable for modeling rotating machinery, motor drives, and other electromechanical interactions (Watthewaduge et al., 2020). In many cases, these problems are posed in terms of Maxwell’s equations – Gauss’s laws, Faraday’s law, and Ampère’s law with Maxwell’s correction—coupled with constitutive relations that govern how electric and magnetic fields interact with charges and currents (see Supplementary Section 2.1). Analytical solutions to these coupled partial differential equations (PDEs) are scarce. Moreover, computing electromagnetic torque from first principles requires derivation through the Lorentz force density or, more conveniently, the Maxwell stress tensor, yielding surface integral formulations over the machine geometry. Popular discretization methods, the finite difference method (FDM) and even the finite element method (FEM), are used to obtain point-wise or piece-wise linear estimates over fine grids or meshed domains of interest (Roubache et al., 2018). Although such numerical approximations have been explored for some time, rapid progress has been seen in stator–rotor coupled simulations, with novel boundary treatments at material interfaces and continual refinements in solver algorithms enhancing computational reliability.

Solving field problems with both high fidelity and computational tractability has also seen significant attention through the integration of sparse experimental data and nonlinear material modeling. Data assimilation and transfer learning have been shown to be effective in calibrating simulation outputs against limited sensor readings. These approaches exploit iterative solvers’ ability to refine model parameters in the presence of magnetic saturation and hysteresis (Guo et al., 2020). Many variants of efficient simulation architectures have been explored to exploit structure, accuracy, and speed of convergence, including surrogate modeling, mesh optimization, and hardware acceleration via FPGAs or GPUs (Mojlish et al., 2017; Praslicka et al., 2023). However, one of the least explored areas is the rigorous propagation of uncertainty through nonlinear field solvers. While most pipelines assume perfectly known material properties and boundary data, in many practical applications this data comes from regulated and calibrated measurement processes subject to uncertainties pertaining to sensor limitations or the stochastic nature of the physical processes themselves. Given the typical sparsity of validation measurements and the strong nonlinearity of magnetic materials, vanilla simulation pipelines can propagate these errors in an unstable fashion. Bayesian calibration, Monte Carlo methods, and Gaussian process regression offer remedies (Ma et al., 2022; Manfredi and Trinchero, 2022), but their integration with high-fidelity physics-based solvers remains an open challenge that limits industrial-strength field prediction.

#### 3.1.1 Utilizing PINNs to model complex electromagnetic phenomena

PINNs offer a transformative framework for modeling complex electromagnetic phenomena in electrical machines and drives by embedding governing equations, such as Maxwell’s equations, and appropriate boundary conditions directly within the loss function (Bajaj et al., 2023). This integration ensures that the network honors fundamental conservation laws and electromagnetic principles, thereby reducing the non-physical solutions that often plague purely data-driven models. By aligning network outputs with analytical benchmarks and experimental data, PINNs achieve enhanced reliability in simulating field distributions, thermal effects, and other behaviors inherent to electrical drives. The careful selection and tuning of boundary conditions, together with embedded physical laws, provide an intrinsic regularization that mitigates the adverse effects of sparse or noisy measurements, while advances in automatic differentiation streamline the computation of complex derivatives during training.

Data integration is an equally critical consideration, as field simulations often rely on sparse experimental or sensor data that may not fully capture the underlying electromagnetic behavior. PINNs address this challenge by fusing limited but high-quality observational data with physics-based constraints during training, incorporating boundary measurements, sensor outputs, and calibration data to correct for systematic discrepancies inherent in simulation-only models. Techniques such as transfer learning and data assimilation enable generalization across diverse operating conditions (Parekh et al., 2023), creating self-correcting models that refine predictions in real time. Equally important is the handling of material nonlinearity—magnetic saturation and hysteresis challenge traditional numerical methods due to their dynamic and often unpredictable nature. PINNs offer an innovative solution by incorporating differential equations that characterize these nonlinear properties, enabling the network to adaptively learn from iterative feedback during training (Yin et al., 2024; J. Guo et al., 2020). Multi-scale modeling techniques are then integrated to reconcile fine-scale interactions—such as local eddy current losses and microscopic flux variations—with the broader electromagnetic performance of the entire machine, achieved through network architectures that capture both microstructural effects and overall system behavior.

Balancing optimization and computational efficiency represents another central challenge in deploying PINNs. The key lies in harmonizing the data-driven loss function with embedded physics-based constraints to ensure rapid and stable convergence without sacrificing accuracy (Ghattas and Willcox, 2021). By carefully calibrating the weightings between empirical data and governing equations, researchers can reduce error propagation and numerical instability, which are particularly problematic in high-dimensional and multi-scale scenarios. Recent innovations, such as adaptive learning rate schedules, gradient regularization, and surrogate modeling techniques like Physics-Informed Gaussian Process regression, manage computational complexity while maintaining high-fidelity predictions (Huber et al., 2023), and hybrid frameworks combining conventional numerical methods with deep learning further accelerate the design optimization process. Surrogate models also reduce reliance on finely discretized meshes and lengthy numerical solvers, allowing rapid convergence even with coarser discretization (Praslicka et al., 2023). Uncertainty quantification complements these efficiency gains: Bayesian calibration and Monte Carlo simulations embed confidence intervals directly into predicted field values, assessing simulation reliability and clearly identifying areas of potential error (Galetzka et al., 2019; Ma et al., 2022). Together, these advances reconcile high-fidelity electromagnetic simulation with the practical constraints of real-time engineering applications, fostering robust, scalable models for electrical machine design and control.

#### 3.1.2 Transfer learning in electromagnetic analysis

Transfer learning in electromagnetic analysis with PINNs for electrical machines and drives requires the rigorous preservation of Maxwell’s equations and associated boundary conditions throughout the transfer process. By embedding these governing laws within the loss function, the simulation-trained model retains physics consistency during retraining, while explicit Dirichlet and Neumann boundary constraints ensure that fundamental electromagnetic interactions are not compromised under parameter refinement (Bajaj et al., 2023). This yields a robust baseline that remains physically plausible while adapting to new operational conditions. Equally important is evaluating the similarity between source and target tasks: comparative analyses of geometry, material properties, and operating conditions determine the degree of overlap between the simulation domain and real-world configurations, guiding training-data selection and architectural adaptation to prevent overfitting or loss of convergence (Ghattas and Willcox, 2021). Hybrid calibration strategies that combine sparse experimental datasets with large-scale simulation outputs then refine predictions iteratively, balancing accuracy with computational efficiency.

### 3.2 Performance estimation

Performance estimation is the process of quantifying the expected loss of a predictive model when applied to unseen data, serving as a critical metric for validating generalization (Brosch et al., 2021). In the context of electrical machines and drives, this process is particularly challenging due to the inherent nonlinearity, temporal dependencies, and uncertainties present in the system dynamics. Researchers have shown that traditional methods may fall short when dealing with complex motor behaviors and transient phenomena (Kirchgässner et al., 2021). As a result, hybrid approaches that combine first-principles modeling with data-driven corrections are increasingly gaining traction. These techniques aim to overcome limitations related to model mismatches and measurement noise, which are often exacerbated by the limited availability of training data. Overall, robust performance estimation not only requires careful selection of estimation techniques but also a nuanced understanding of the underlying physical processes, ensuring that models remain reliable and physically consistent across varying operating conditions.

#### 3.2.1 Hybrid models for accurate state estimation

A critical component in advancing performance estimation for electrical machines is the development of hybrid models for accurate state estimation. These models integrate traditional physics–based representations with modern ML methods to compensate for unmodelled dynamics and uncertainties. By leveraging fundamental physical laws, hybrid models ensure physical consistency while incorporating data–driven corrections to address phenomena such as flux linkage harmonics and inverter nonlinearities (Brosch et al., 2021). This approach necessitates that the model dynamically adapts to deviations from nominal conditions, thereby enhancing its robustness against noise and measurement errors. In addition, the integration of data–driven elements allow for capturing transient behaviors with greater precision, which is essential in systems where rapid changes can lead to instability. Although promising, this strategy poses challenges in maintaining the balance between fidelity to physical laws and the flexibility of ML adjustments, ultimately impacting the overall accuracy of performance estimation in complex drive systems.

#### 3.2.2 Online parameter identification

In parallel, online parameter identification is pivotal for real–time performance estimation in electrical drives. In these applications, the system must continuously update its state estimates and model parameters as new data become available, ensuring that the model remains responsive to changing operating conditions. Speed and responsiveness are therefore crucial, as delays in adapting to transient changes can lead to inaccurate state estimations. Moreover, online methods must be robust to noise, given that measurement uncertainties and disturbances are inherent in real–world applications (Zhu et al., 2021). Computational efficiency is equally important, as the algorithms employed need to operate within stringent time constraints without compromising accuracy. These methods often rely on recursive or iterative algorithms that adjust parameters in real time, but the challenge remains to balance rapid adaptation with the prevention of error propagation—a common pitfall when the training data are sparse or highly variable (Cerqueira et al., 2020). Consequently, ongoing research in online parameter identification focuses on optimizing these tradeoffs to enhance the overall reliability of ML applications in electrical machine control.

#### 3.2.3 Offline parameter identification

Offline parameter identification, by contrast, leverages accumulated historical data to refine and calibrate the underlying models over longer time horizons. This approach facilitates deep calibration, allowing for computationally intensive analyses that yield improved long–term accuracy. By periodically recalibrating the model, offline methods can address systematic discrepancies between predicted and actual performance, effectively incorporating error analysis and correction into the estimation process (Wallscheid, 2021). Such recalibration is particularly beneficial in environments where operating conditions evolve slowly, and short–term fluctuations may obscure longer–term trends. Moreover, offline identification supports model refinement by integrating insights derived from comprehensive data sets, which can lead to a more robust representation of the machine’s behavior under varied conditions. However, this process is not without challenges; ensuring that the historical data accurately reflect current operating conditions requires careful selection and validation of the training data. As such, while offline parameter identification offers a pathway to enhanced accuracy and reliability, it must be implemented in conjunction with strategies that continuously monitor and adjust for discrepancies over time.

### 3.3 ML-driven design and optimization methods with physical constraints

**Figure 5:**
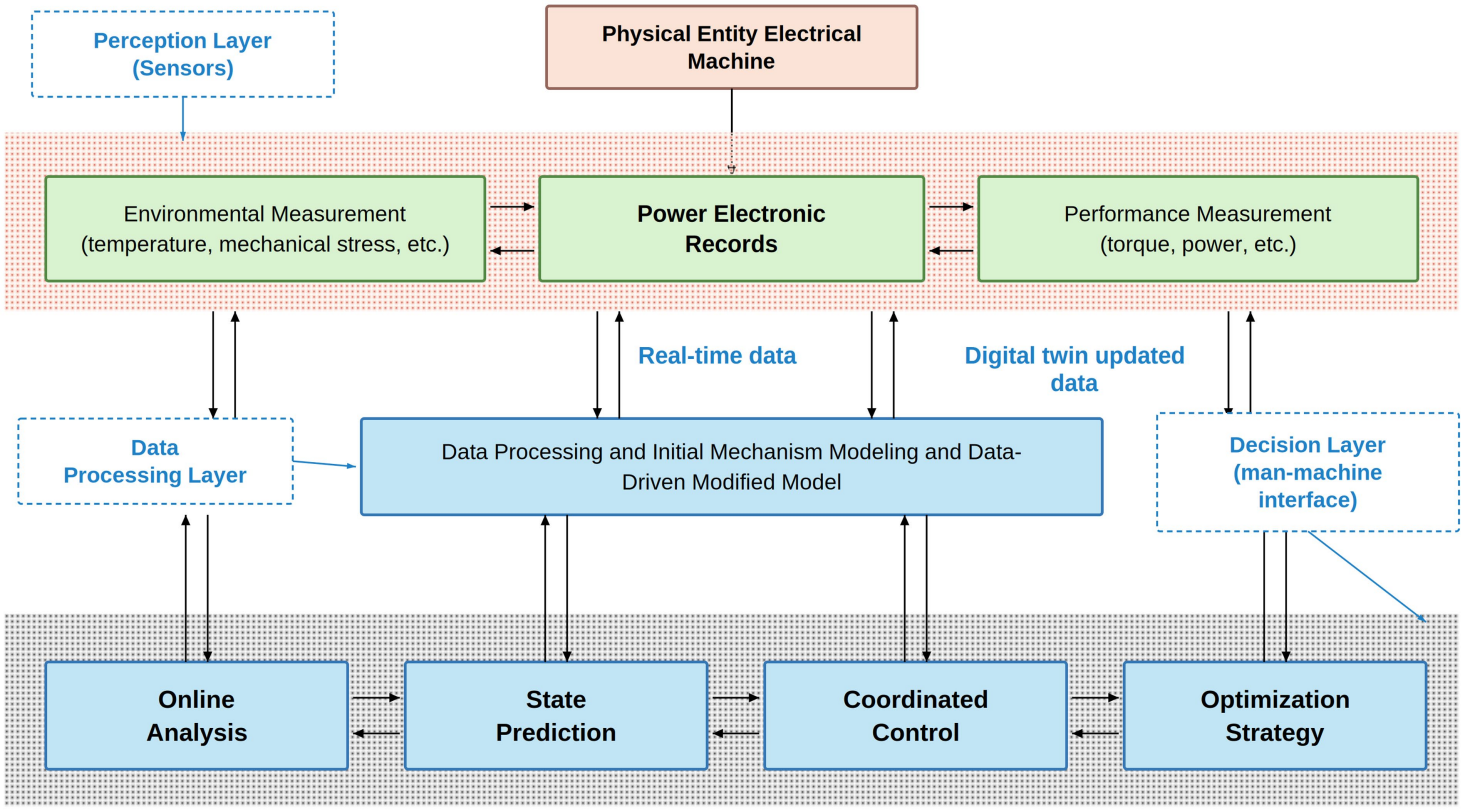
PIML for electrical machines and drives: performance, control, optimization, and digital twins.

Three pivotal methods underpin the design and optimization of electrical machines and drives under the PIML umbrella: deterministic (gradient-based) optimization, stochastic (metaheuristic) optimization, and surrogate modeling using ML and ANN approaches (Asef and Vagg, 2024). Deterministic optimization leverages gradient information to systematically refine continuous design variables while embedding physical constraints, ensuring rapid convergence and adherence to system physics–particularly suited to differentiable models requiring fine-tuned performance adjustments (see Supplementary Section 1.4) (Nghiem et al., 2023). Stochastic optimization complements this by employing metaheuristics such as genetic algorithms and physics-informed Bayesian optimization to explore the design space globally, an approach especially valuable when the optimization landscape is non-convex, discontinuous, or riddled with local minima that purely gradient-based methods would overlook (Antonion et al., 2024; Zhao et al., 2024). Surrogate modeling, in turn, constructs computationally efficient ML-based approximations of complex physical simulations, integrating data-driven predictions with physical constraints to deliver rapid performance assessments when experimental data are scarce or high-fidelity simulations are prohibitively expensive (Tahkola et al., 2020). Together, these methods bridge theoretical simulation and practical application, fostering robust, physically consistent design frameworks for next-generation electrical machines and drives. Figure 5 illustrates PIML applications spanning field simulation, performance estimation, design optimization, neural control, digital-twin calibration, and deployment.

### 3.4 Neural networks for the control of electrical machines

#### 3.4.1 Intelligent control strategies for drives

PIML has been increasingly integrated into model-based control strategies for electric drives, most notably Model Predictive Control (MPC). PIML-MPC embeds detailed physics-based models within an optimization framework to forecast system behavior over a receding horizon, enabling real-time adjustment of control actions while explicitly managing constraints. By incorporating ML techniques informed by underlying physical principles, this approach maintains predictive accuracy even under parameter uncertainty and external disturbances (Xue et al., 2023), and recent advances in weighting factor calculation and harmonic distortion optimization have further improved computational tractability and robustness (Elmorshedy et al., 2021). Reinforcement learning (RL)–based control offers a complementary, model-free strategy in which optimal control policies are learned iteratively through direct interaction with the drive system, adapting to nonlinearities and uncertainties via experience-driven improvements (Farah et al., 2023). When augmented with physics-informed constraints, RL controllers gain enhanced robustness and adaptability, balancing exploration and exploitation in dynamic environments (Book et al., 2021), and have shown promising results for permanent magnet synchronous and DC motors where conventional controllers struggle (Schenke et al., 2020). Computational complexity and sim-to-real transfer remain open challenges, but ongoing refinements continue to position RL-based control as a viable solution for next-generation drives (Kazemikia, 2024).

A third strategy, hybrid intelligent control, fuses the strengths of neural networks, fuzzy logic, and classical control by combining Adaptive Neuro-Fuzzy Inference Systems (ANFIS) with data-driven methodologies. This approach capitalizes on ANFIS’s ability to approximate nonlinear relationships and adapt to changing system dynamics, improving parameter adaptation and fault tolerance in electric drives (Parra et al., 2018). By merging traditional model knowledge with ML insights, hybrid intelligent control mitigates the limitations of singular approaches and delivers gains in energy management, dynamic performance, and overall efficiency (Rajasekar and Ashok, 2024; Nyangon, 2021a), with demonstrated superiority in torque vectoring, regenerative braking, and real-time optimization in multi-motor setups.

#### 3.4.2 Adaptive controllers based on PIML models

Adaptive controllers dynamically adjust their parameters in response to real-time performance and environmental uncertainties, compensating for model discrepancies, external disturbances, and time-varying dynamics (Nyangon, 2025a; Nghiem et al., 2023). Recent advances in PIML have invigorated this domain by embedding physical constraints into neural network frameworks, enabling more accurate and robust modeling of complex systems. For electrical machines and drives, PIML-based adaptive controllers harness the ability of neural networks to approximate highly nonlinear functions while incorporating governing physical laws into the training process (Hanover et al., 2022), significantly enhancing stability under variable load conditions and unpredictable disturbances while improving precision in trajectory tracking. Their integration with established methods—such as iterative learning control (ILC) and MPC—further reinforces robustness by compensating for systematic tracking errors and model uncertainties. In practice, combining neural-network-assisted control with physics-based constraints mitigates the adverse effects of nonlinearities, hysteresis, and measurement noise typical of electrical drive systems (Feng et al., 2020; Yang et al., 2019), permitting rapid convergence toward the desired operating regime while keeping error propagation controlled even under imperfect boundary conditions or varying operational demands (Nan and Hutter, 2024; Nghiem et al., 2023). This dual capability of maintaining robust performance while facilitating transfer learning across distinct dynamic environments—from robotic motion control to high-precision tracking in piezoelectric actuators—underscores the potential of PIML-based adaptive controllers as industrial-grade solutions, with simulation studies and experimental validations highlighting their suitability for complex, safety-critical applications.

### 3.5 Physics-informed learning for next-generation digital twins

**Figure 6:**
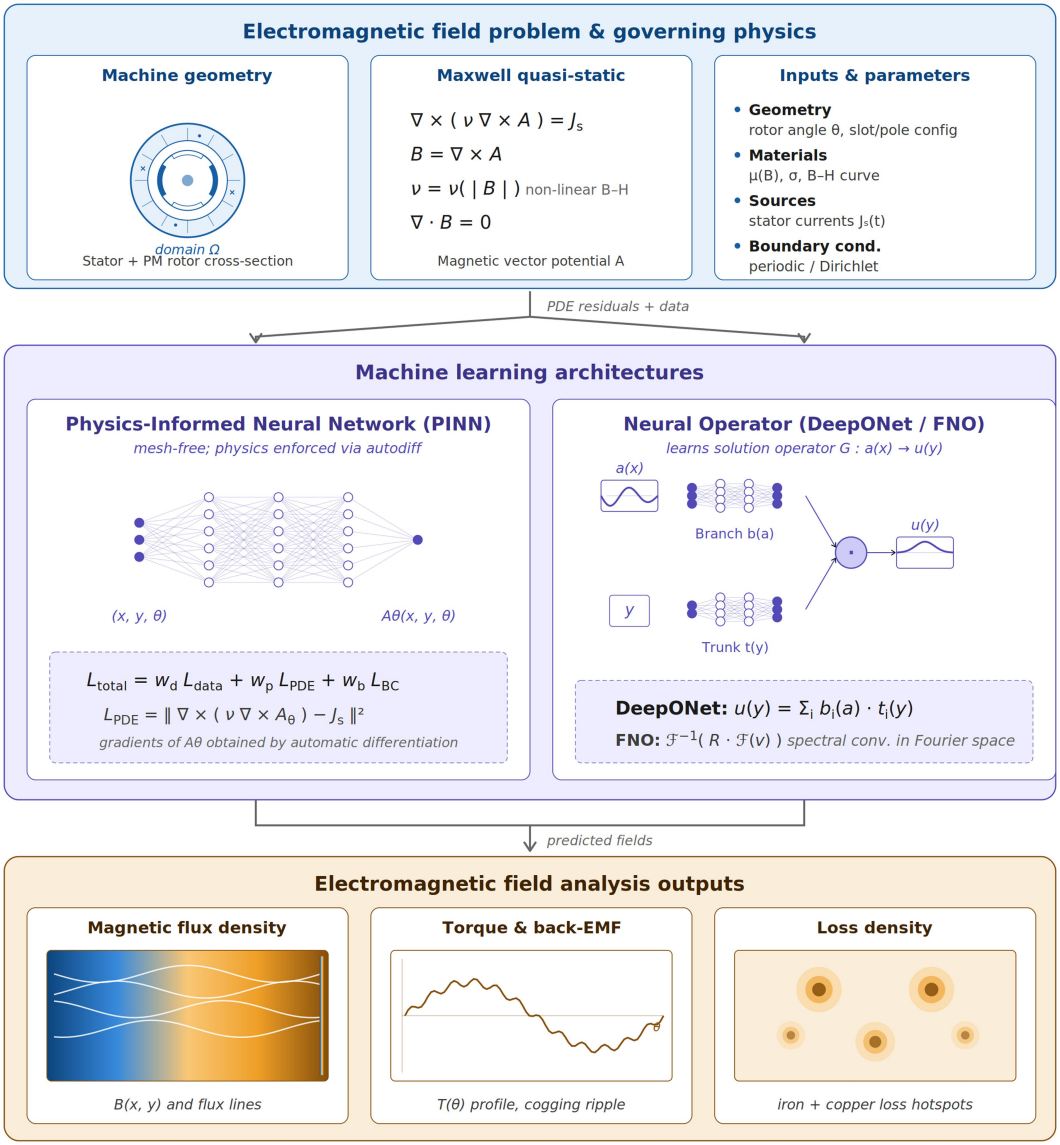
The four key components of the digital twin modeling framework for electrical drive systems.

Digital twins are virtual replicas of physical systems that mirror real-time behavior, enabling precise simulation and continuous monitoring of electrical machines and drives (Bouzid et al., 2020). Integration with existing control systems follows a systematic four-part architecture comprising the physical entity, a perception layer for comprehensive data acquisition, a middle layer for data processing and simulation, and a decision layer for human–computer interaction (Liu et al., 2022) (see Figure 6). The perception layer gathers extensive operational data—from motion drive metrics to status signals—while the middle layer synthesizes this information into robust simulation models using multidisciplinary fundamentals such as finite element analysis and data-driven approaches. The decision layer then maps digital data to actionable insights, facilitating closed-loop feedback and timely controller intervention. Recent studies show that embedding digital twins within existing controllers—as demonstrated in power electronic converters and rotating machinery—significantly enhances real-time diagnostics and fault prediction (Cheruku et al., 2025). When integrated with Manufacturing Execution Systems (MES), digital twins further trigger automated actions on physical equipment, ensuring synchronized operation between digital simulations and real-world processes (Nyangon, 2025b; Negri et al., 2020). This integrated approach improves real-time monitoring precision and mitigates the propagation of uncertainties arising from sensor inaccuracies, reinforcing the overall stability and resilience of control systems.

#### 3.5.1 Real-time monitoring and diagnostics

Real-time monitoring and diagnostics, underpinned by PIML, offer a transformative approach to managing electrical machines and drives. Digital twins serve as high-fidelity virtual replicas that enable dynamic simulation and continuous evaluation of complex electromagnetic and thermal behaviors, supporting fault detection, design optimization, and predictive maintenance through the integration of data-driven models with finite element methods and probabilistic frameworks (Cheruku et al., 2025; Liu et al., 2022). This convergence allows rapid, precise characterization of machine states even when physical measurements are challenging or incomplete—capabilities crucial in high-demand industrial environments where timely intervention can avert catastrophic failures (Milton et al., 2020). In practice, digital twin frameworks are embedded within controller architectures to establish closed-loop feedback that synchronizes virtual simulations with the physical behavior of electrical machines, while combinatorial data synthesis strategies improve the predictive performance of reduced-order models and yield higher fault detection accuracy (Cheruku et al., 2025; Nyangon, 2025b). Augmented reality tools further support interactive human intervention through real-time alerts and visualizations, and partitioning digital twin models into discrete control layers enables targeted analysis of power electronic converters and rotating machinery, ensuring that both global system behavior and local fault conditions are comprehensively monitored (Bouzid et al., 2020; Diz et al., 2023). This multi-layered approach mitigates measurement-error propagation and enhances scalability across diverse industrial scenarios.

#### 3.5.2 Comparative analysis of traditional ML and PIML approaches

Traditional machine learning methods – linear and logistic regression, decision trees, random forests, and support vector machines (SVMs) – have long served as valuable tools for enhancing electrical machine and drive performance, relying on statistical inference and data-driven optimization to model relationships between input variables and system behavior. Linear and logistic regression offer computational simplicity and interpretability, decision trees and random forests capture non-linear interactions efficiently (Gawande, 2024), and SVMs deliver robust performance on complex decision boundaries, enabling precise fault diagnosis and predictive control (Wu et al., 2024). However, these methods are inherently limited by their inability to incorporate explicit physical laws governing system dynamics, and their performance often diminishes when confronted with the high-dimensional, non-linear processes typical of modern drives, particularly when data are scarce or noisy. PIML methods—including PINNs, PIGNNs, Deep Operator Networks (DeepONets), FNOs, and PIGPs – represent a paradigm shift by embedding first-principles knowledge directly into the learning process, delivering enhanced accuracy and robustness in systems exhibiting complex, non-linear dynamics (Zhao et al., 2022) (See Table 4). PINNs and PIGNNs integrate differential equation solvers with deep learning to ensure adherence to underlying physical laws, while DeepONets and FNOs excel at learning operators that map infinite-dimensional spaces with improved generalization (Sharma et al., 2023), and PIGPs provide a probabilistic framework for uncertainty quantification under sparse data. This hybrid approach mitigates the limitations of traditional methods by reducing error propagation and ensuring model consistency across varying operating conditions (Zideh et al., 2024; Huber et al., 2023; Parekh et al., 2023), with demonstrated superiority in capturing transient behaviors and accommodating measurement uncertainties – offering a robust alternative for real-time control and fault diagnostics in advanced electrical drive systems.

**Table 4:** Comparative analysis of traditional ML vs. PIML methods for electrical machines drives.

Method	Description	Key feature and strength	Application to electrical machines drives	Method type
Linear regression	Models linear relationships between input features and a continuous output.	Simplicity and high interpretability Low computational cost Effective for linearly-behaving datasets	Predicting performance metrics (e.g., torque, speed, consumption) Parameter estimation and calibration	Traditional ML
Logistic regression	Uses a logistic function to model binary or multi-class outcomes.	Straightforward implementation Probabilistic outputs Computationally efficient	Fault detection and classification Operational state monitoring	Traditional ML
Decision trees	Tree-based models that split data based on feature thresholds to make predictions.	High interpretability Captures non-linear relationships Minimal data pre-processing required	Diagnostics and failure mode detection Performance categorization	Traditional ML
Random forests	An ensemble of decision trees that aggregates predictions to improve accuracy.	Robustness against overfitting Effective with high-dimensional data Improved predictive accuracy	Predictive maintenance Reliability assessment Optimization of machine performance	Traditional ML
Support vector machines (SVMs)	Finds the optimal hyperplane for classification or regression tasks.	Effective in high-dimensional spaces Versatile with kernel functions Robust to outliers	Fault classification Anomaly detection Control system tuning	Traditional ML
PINNs	Neural networks that incorporate physical laws (e.g., differential equations) into loss function during training.	Enforces physical consistency Enhanced optimization and extrapolation Effective with sparse data	Modeling electromagnetic fields Thermal analysis Dynamic behavior simulation	PIML
PGNNs	Neural networks that integrate physics-based constraints during training for improved model behavior.	Balances data-driven learning with physics insights Improved interpretability Better extrapolation capabilities	Real-time monitoring System control and performance optimization Adaptive behavior prediction	PIML
Deep Operator Networks (DeepONets)	Networks designed to learn operators that map between infinite-dimensional function spaces.	Learns complex system operators Optimizes to unseen conditions Operates at an operator level rather than pointwise predictions	Control operator design Dynamic simulation Modeling non-linear behaviors	PIML
FNOs	Neural operators that leverage Fourier transform techniques to learn mappings in function spaces efficiently.	Efficient handling of high-dimensional data Fast training times Captures long-range dependencies in spatial data	Fast simulation of spatially distributed phenomena Real-time control applications	PIML
Physics-informed Gaussian processes	Gaussian process models enhanced with physics-based priors to improve predictions and quantify uncertainty.	Probabilistic predictions with built-in uncertainty quantification Integrates physical constraints for improved robustness Flexible non-parametric modeling.	Fault detection with uncertainty analysis Reliability assessments Predictive maintenance in environments with limited data	PIML

## 4 Challenges and future perspectives

Industry 4.0 is transforming the manufacturing landscape through the integration of the Internet of Things (IoT), digital twins, and advanced data analytics into industrial systems (Nyangon, 2025b; Ji and Abdoli, 2023). In the electrical machines and drives market, this paradigm shift enables real-time monitoring, enhanced diagnostic precision, and predictive maintenance through the convergence of physical and digital realms, with PIML embedding physical laws into data-driven models to enhance reliability and interpretability. However, the implementation of PIML in this context is fraught with challenges. Chief among these is the computational complexity associated with modeling high-dimensional, nonlinear phenomena in electrical systems, which often demands sophisticated numerical solvers and extensive computational resources. Variability and uncertainty in operational data, coupled with context-dependent calibration issues, further impede the development of universally robust models, while the limited availability of high-fidelity training data and algorithmic sensitivity to noisy inputs frequently result in unstable predictions—hindering industrial adoption (Dalzochio et al., 2020).

Future expectations for PIML in the electrical machines and drives sector center on overcoming these computational and practical challenges through algorithmic efficiency and sustained research effort. Advances in numerical methods and parallel computing in the Industry 4.0 era are expected to mitigate the scalability issues that currently constrain high-dimensional, nonlinear modeling (Çınar et al., 2020). Equally critical is the translation of theoretical models into industrial applications, where iterative validation against real-world data is essential to address uncertainties and data imperfections, bridging the gap between academic innovation and practical implementation to refine PIML frameworks for reliable performance in dynamic operational environments. Cross-disciplinary collaboration among experts in control engineering, computational physics, and data analytics will further drive the creation of resilient, adaptable models. Together, these improvements are poised to reinforce the transformative impact of Industry 4.0 on predictive maintenance, system optimization, dynamic behavior simulation, real-time monitoring, controller design, nonlinear modeling, and fault detection with uncertainty analysis. With coordinated efforts to reconcile theoretical advances with empirical challenges, PIML is anticipated to deliver significant gains in efficiency, reliability, and diagnostic performance in the Industry 4.0 era.

### 4.1 Strengths, limitations, and failure modes of PINNs, PIGNNs, and neural operators

PINNs remain the most widely adopted PIML approach for electrical machine modeling, owing to their elegant integration of governing PDEs as soft constraints in the loss function and their mesh-free formulation, which makes them attractive for irregular stator–rotor geometries and inverse problems involving sparse measurements (Raissi et al., 2019; Bajaj et al., 2023). Their universal function approximation capability and compatibility with automatic differentiation enable rapid prototyping across varied boundary conditions. However, PINNs exhibit well-documented failure modes that limit industrial deployment. They suffer from spectral bias—a tendency to fit low-frequency components first and struggle with high-frequency electromagnetic phenomena such as slot harmonics or fast switching transients (Wang et al., 2022). Training pathologies arise from competing loss terms (data, PDE residual, boundary, and initial conditions), where gradient imbalance can cause convergence to trivial or unphysical solutions, particularly in stiff or multi-scale regimes characteristic of saturated magnetic materials. PINNs also generalize poorly across geometries: a model trained for one motor topology must typically be retrained for another, and convergence degrades sharply as domain complexity increases (Karniadakis et al., 2021).

PIGNNs and neural operator frameworks address several of these shortcomings, though with their own trade-offs. PIGNNs natively handle the unstructured meshes used in finite-element electromagnetic analysis and naturally encode topological relationships between stator slots, rotor poles, and air-gap regions, making them well-suited to fault diagnosis and parameter estimation across heterogeneous machine designs (Zhao et al., 2022). Their principal limitations are over-smoothing as message-passing depth increases and difficulty propagating long-range field interactions across large meshes. Neural operators, including DeepONets and FNOs, learn mappings between infinite-dimensional function spaces, enabling a single trained model to generalize across families of boundary conditions, excitation waveforms, or material parameters without retraining, which dramatically accelerates design-space exploration (Lu et al., 2021; Sharma et al., 2023). FNOs deliver exceptional speed on periodic structures but are constrained to regular grids, limiting their direct applicability to complex motor geometries; DeepONets are mesh-agnostic but require substantial paired training data and can struggle with sharp discontinuities such as those at iron–air interfaces. Hybrid approaches, combining neural operators for global field prediction with PINN-style residuals for local enforcement, or coupling PIGNNs with Physics-Informed Gaussian Processes for uncertainty quantification–are emerging as a pragmatic path to mitigate individual weaknesses while preserving physical consistency (Huber et al., 2023; Zideh et al., 2024). Table 5 summarizes the strengths and trade-offs of major PIML approaches across four key deployment dimensions: scalability, data efficiency, robustness, and real-time deployability.

**Table 5:** Comparison of major PIML approaches.

Dimension	PINNs	PIGNNs	DeepONets	FNOs	PIGPs
Scalability	Limited–degrades on high-dimensional, multi-scale problems; spectral bias hampers high-frequency phenomena (slot harmonics, fast switching)	Good–native handling of unstructured FE meshes; scales with graph size but suffers over-smoothing at depth (Zhao et al., 2022)	Very good–operator mappings generalize across boundary conditions and excitations without retraining (Lu et al., 2021)	Excellent on regular grids–spectral convolutions yield up to ~100 ms/sample versus hours for FEM (Parekh et al., 2023; Sharma et al., 2023)	Limited–O(N^3^) inference cost; struggles beyond moderate dataset sizes (Huber et al., 2023)
Data efficiency	High–PDE residuals enable training with sparse or no labeled data (Bajaj et al., 2023)	Moderate–High -topology and physics constraints reduce data demand	Lower–requires substantial paired (input, output) function data	Lower–needs dense training data on regular grids	Excellent–Bayesian framework natively handles sparse, noisy measurements (Zideh et al., 2024)
Robustness	Moderate–sensitive to loss-term weighting; gradient imbalance can yield trivial or unphysical solutions in stiff/saturated regimes.	Good–encodes stator–rotor topology; resilient across heterogeneous geometries (Zhao et al., 2022)	Moderate–struggles with sharp discontinuities at iron–air interfaces (Sharma et al., 2023)	Limited–confined to periodic/regular geometries; poor on irregular motor cross-sections	Excellent–built-in uncertainty quantification via posterior variance (Huber et al., 2023; Zideh et al., 2024)
Real-time deployability	Moderate–fast inference once trained, but retraining typically required per new geometry or boundary condition (Karniadakis et al., 2021)	Good–efficient graph inference; well-suited to online fault diagnosis (Zhao et al., 2022)	Excellent–a single trained operator replaces many simulations across the design space (Lu et al., 2021)	Excellent–fastest inference among PIML approaches; ideal for digital-twin loops on regular topologies (Parekh et al., 2023)	Limited–inference cost grows with training set; better for offline calibration than real-time control

No single architecture dominates all four dimensions. PINNs offer the best data efficiency for inverse problems but pay for it in scalability and training stability. PIGNNs strike the most balanced profile for industrial motor geometries. DeepONets and FNOs excel at scalability and real-time deployment but require richer training corpora and falter on sharp material interfaces. PIGPs remain the strongest choice when uncertainty quantification is non-negotiable, despite their cubic cost. Hybrid pipelines–e.g., FNO-based global field prediction with PINN-style local residuals, or PIGNN backbones with PIGP heads for confidence intervals–are emerging as the pragmatic frontier (Huber et al., 2023; Zideh et al., 2024).

### 4.2 Computational complexity and scalability

In the context of Industry 4.0, PIML is set to transform the design and control of electrical machines and drives by dramatically reducing computational complexity. Recent studies have demonstrated that integrating physical principles directly into ML frameworks not only enhances prediction accuracy but also leads to substantial reductions in processing times. For instance, the use of physics-informed Bayesian optimization coupled with a maximum entropy sampling algorithm (PIBO-MESA) has yielded processing time reductions of approximately 45% compared with stochastic methods such as Non-Dominated Sorting Genetic Algorithm II (NSGA-II) (Asef and Vagg, 2024). Similarly, employing a multi-branch deep neural network has reduced simulation times from several hours—typically in the range of three to 5 hrs—to roughly 100 milliseconds per sample when replacing traditional finite element simulations (Parekh et al., 2023). In an Industry 4.0 framework, where rapid adaptability and real-time monitoring are essential, such improvements in computational efficiency are critical for both design optimization and operational control.

Beyond computational speed, PIML methods substantially contribute to scalability in complex industrial applications. The ability to handle large datasets and complex, high-dimensional models is inherent in many PIML architectures, which combine physics-based constraints with the adaptive learning capabilities of neural networks. This dual approach not only mitigates the burden of traditional simulation techniques but also ensures that models remain robust when scaling from laboratory prototypes to full industrial systems. Integration with digital twin technologies further exemplifies this scalability. A digital twin of an electrical machine or drive assets can incorporate PIML algorithms to simulate and predict the machine or drive behavior in real time, enabling predictive maintenance and adaptive control strategies across large-scale production systems (Cheruku et al., 2025). Such systems are designed to operate seamlessly within cyber-physical frameworks, ensuring that improvements in computational performance directly translate into enhanced system reliability and operational efficiency. As these models are refined, they promise to underpin scalable solutions that are adaptable to a wide range of applications, from traction motor design in electric vehicles to power electronic converter optimization.

Despite these advances, several challenges and future expectations remain regarding computational complexity and scalability. One significant challenge is the inherent trade-off between model complexity and computational efficiency. While deeper and more sophisticated PIML models can capture intricate physical phenomena more accurately, they may also demand increased computational resources. This raises concerns about the generalization of such models when applied to very large or complex systems. Moreover, current studies often rely on specific baseline comparisons, and there is a pressing need for standardized benchmarking procedures that can reliably assess scalability across diverse applications. Future research should focus on testing PIML approaches on larger and more heterogeneous datasets, ensuring that performance improvements observed in controlled experiments can be replicated in full-scale industrial environments. Additionally, further integration of PIML with digital twin platforms is anticipated to facilitate comprehensive, real-time monitoring and control, thus bolstering the overall efficiency and resilience of electrical machine systems in Industry 4.0 (Zhao et al., 2022). More prosaically, while PIML offers promising improvements in computational efficiency and scalability, addressing these challenges will be crucial to fully realize its potential in transforming industrial practices.

### 4.3 Bridging the gap between theory and industrial applications

PIML is an emerging pivotal method for bridging the longstanding gap between theoretical models and industrial applications in electrical machines and drives within the Industry 4.0 framework. By integrating rigorous physical laws directly into data-driven models, PIML enables the development of more reliable and computationally efficient tools that are capable of real-time operation. Current theoretical approaches—such as finite element analysis (FEA) and finite difference methods (FDM)—are commonly employed to simulate electromagnetic fields and thermal behavior, as well as discretize and solve partial differential equations governing machine operation. However, these methods often suffer from prohibitive computational times and a lack of robustness when confronted with real-world uncertainties—limitations that hinder their practical deployment. For example, conventional finite element simulations, while highly accurate, are computationally intensive and poorly suited for dynamic industrial environments, whereas PIML methods can drastically reduce simulation times and incorporate uncertainties in boundary conditions and material properties. This fusion of physics-based reasoning with adaptive ML not only enhances predictive accuracy and reliability but also facilitates the seamless integration of digital twins into existing control systems, thereby supporting scalable and adaptive maintenance strategies. Moreover, hybrid models that couple physical constraints with statistical learning are showing promise in improving model generalization across diverse operational scenarios. As noted by Liu et al. (2022) and Parekh et al. (2023), such frameworks offer a viable pathway to overcome the disconnect between laboratory-based theoretical advancements and the practical demands of industrial-scale applications, paving the way for optimized design and robust, real-time control in the rapidly evolving industrial landscape.

### 4.4 Future directions for integrating PIML in electrical systems

The trajectory of PIML in electrical systems hinges on cross-disciplinary collaboration, and several lessons have crystallized from recent progress. First, embedding domain expertise directly into learning models, through sustained partnerships between ML researchers and specialists in physics, engineering, and mathematics, consistently yields more realistic predictions and stronger interpretability than purely data-driven alternatives, with transferable benefits across fluid mechanics, chemical engineering, and additive manufacturing (Wu et al., 2024; Sharma et al., 2023). Second, hybrid architectures that pair classical simulation techniques with data-driven learners outperform either paradigm in isolation: they deliver modular, stable, and accurate frameworks capable of capturing phenomena beyond the reach of single methodologies while enabling advanced system identification and control (Hanover et al., 2022). The corresponding best practice is to treat physics-based solvers and neural components as complementary modules rather than competitors, using each where its inductive bias is strongest. Third, reproducibility and benchmarking remain bottlenecks; shared databases, standardized experimental protocols, and common evaluation metrics are essential for systematic validation, provided that data structure, security, and interoperability are addressed under appropriate ethical and legal standards (Torchio et al., 2025; Cerqueira et al., 2020).

Building on these foundations, future progress will depend on disciplined algorithm development and rigorous uncertainty management. Innovations driven by the fusion of computer science, applied mathematics, and domain expertise are needed to handle noisy, sparse, and high-dimensional data, with measurable gains in optimization, convergence, and scalability now an explicit design target (Moradi et al., 2023). A parallel best practice is to embed uncertainty quantification and transparency from the outset rather than retrofitting them: Bayesian neural networks, Monte Carlo dropout, and probabilistic surrogates deliver predictions alongside clear insight into the underlying physical processes, which is indispensable for safety-critical electrical drive and digital-twin applications (Rezazadeh et al., 2025; Bharadwaja et al., 2022; Galetzka et al., 2019). Finally, durable progress requires investment in educational and collaborative infrastructure—interdisciplinary workshops, joint research projects, and cross-training initiatives—that prepares a new generation fluent in both ML and the physical sciences. Curricula that incorporate PINNs, digital twins, surrogate modeling, Bayesian optimization, multi-physics and multi-scale simulation, and adaptive intelligent control systems offer immersive experiences that seed long-term collaborative ecosystems (Nyangon and Akintunde, 2024). Together, these lessons and practices bridge theoretical and practical gaps, accelerating the dissemination of cutting-edge PIML across the electrical machines and drives industry and positioning the field for sustained, transformative innovation.

## 5 Conclusion

Recent advances in physics-informed machine learning (PIML) are revolutionizing the design and optimization of electrical machines and drives. This state-of-the-art review demonstrates that integrating physical laws directly into ML frameworks enhances modeling accuracy while significantly reducing computational costs. Methodologies such as physics-informed neural networks (PINNs) and hybrid data–physics approaches offer robust solutions to the challenges of modeling complex electromagnetic phenomena and multi-scale interactions inherent in electrical machines. By embedding Maxwell’s equations and other governing principles into the learning process, these methods achieve higher fidelity in simulations and more reliable performance predictions. The concurrent shift toward mesh-free and domain decomposition techniques, along with the use of Bayesian optimization, highlights a clear trend toward adaptable, efficient design tools capable of operating under real-time conditions – heralding a new era of electrical machine design that is both data-efficient and physically consistent (Asef and Vagg, 2024; Nyangon, 2021b).

Building on this integration, recent research has successfully addressed key challenges such as data sparsity and the need for rapid design iteration. The developed algorithms not only improve computational efficiency but also expand the range of applicable operating conditions by effectively capturing nonlinearity and hysteresis effects. PINNs, for instance, have enabled the replacement of traditional, computationally intensive finite element methods in many electromagnetic problems, while seamlessly incorporating multi-scale models that provide comprehensive insights from local field variations to overall machine behavior. These approaches also support improved parameter estimation and fault diagnostics—critical for enhancing the reliability of electrical drives under dynamic operating conditions (Cheruku et al., 2025). Collectively, the evidence indicates that a unified framework combining physics and data-driven learning can robustly address longstanding modeling challenges, reducing design time while improving efficiency, torque, and noise performance across applications ranging from automotive drives to advanced industrial machinery.

Future research should concentrate on refining the integration of multi-physics constraints into neural architectures to reduce training complexity and improve convergence, while developing adaptive methods that dynamically adjust to real-time variations in system parameters for more resilient predictive maintenance and control frameworks (Nyangon, 2025a; Nyangon, 2021a). Advances in hardware acceleration and parallel computing will further enable real-time industrial deployment under rigorous adherence to physical laws, and the continued fusion of data-driven techniques with traditional numerical simulations promises cost-effective, scalable design optimization. As these methods mature, PIML is anticipated to become a cornerstone technology for next-generation electrical machine design, ultimately contributing to more sustainable, high-performance energy systems.

## Data Availability

The original contributions presented in the study are included in the article/Supplementary material, further inquiries can be directed to the corresponding author/s.
