# Supplementary material for: Hybrid physics-informed artificial intelligence for high-fidelity modeling and optimization of electrical systems
Source: Front Artif Intell. 2026 May 28;9:1751785. doi: 10.3389/frai.2026.1751785 (PMC13253959; doi:10.3389/frai.2026.1751785)
Supplement: Supplementary file 1 [file Data_Sheet_1.pdf]

Hybrid Physics-Informed Artificial Intelligence for High-Fidelity Modeling and Optimization of Electrical Systems

Supplementary Materials

1. PIML and Operator Learning for Electromagnetic Field Analysis of Electrical Machines

1.1. Enhanced PINN Architectures

Recent developments in enhanced PINNs have transformed these models into more robust and efficient tools for tackling complex engineering problems (Nyangon, 2025a). A major evolution has been the hybridization of traditional PINNs with ELMs, which substitute iterative gradient-based updates with a single-step least-squares solution. This hybrid approach, as demonstrated by Joshi et al. (2024) and Khademi & Dufour (2024), significantly reduces training times while maintaining high accuracy, especially in linear or mildly nonlinear systems. Additionally, theory-constrained PINNs have emerged by embedding explicit physical laws, such as first-order shear deformation theory, directly into the loss function (Zhang et al., 2023). This innovation ensures that predictions adhere more closely to established physical behavior, thus narrowing the solution space and enhancing reliability in scenarios with complex boundary conditions. The integration of physics-based regularization within the training loss further mitigates overfitting and improves model fidelity (Nyangon, 2025a).

Furthermore, advancements in loss function design and optimization have played a crucial role in enhancing PINN performance. Multi-objective loss functions now balance data-driven and physics-based terms through adaptive weighting and dynamic tuning, which alleviates issues like gradient imbalances during training. These improvements not only accelerate convergence but also ensure that the network effectively learns both empirical data and embedded physical constraints. Figure 1 shows enhanced PINNs combining PINN–ELM architecture and physics module with multi-objective loss—Pareto optimization, adaptive weighting, Lagrangian methods—producing solutions, parameter inference, uncertainty, and downstream surrogates. Finally, scalability and robustness have been markedly improved by leveraging domain decomposition techniques, such as those found in Finite Basis PINNs (FBPINNs) (Moseley et al., 2023). A simple FBPINNs formulation augments a neural network's representation with Fourier basis functions. For example, one may represent the solution $u(x)$ as

$$u(x) \approx \sum_{k=1}^{K} [a_k \cos(2\pi\omega_k x) + b_k \sin(2\pi\omega_k x)] + f_\theta(x),$$

where:

- The Fourier series component $\sum_{k=1}^{K} [a_k \cos(2\pi\omega_k x) + b_k \sin(2\pi\omega_k x)]$ captures the global, low-frequency behavior.

- $f_\theta(x)$ is a neural network (with parameters θ) that accounts for local high-frequency corrections or residual features.
- a_k, b_k are Fourier coefficients and ω_k are predefined frequencies.

The training loss then combines data fitting with physics constraints:

$$\mathcal{L}(\theta, a, b) = \frac{1}{N}\sum_{i=1}^{N} | u(x_i) - \sum_{k=1}^{K}[a_k \cos(2\pi\omega_k x_i) + b_k \sin(2\pi\omega_k x_i)] - f_\theta(x_i) |_2 + \lambda \frac{1}{M}\sum_{j=1}^{M} | \mathcal{N}\left[\sum_{k=1}^{K}\left[a_k \cos\left(2\pi\omega_k x_j\right) + b_k \sin\left(2\pi\omega_k x_j\right)\right] + f_\theta\left(x_j\right)\right] |_2.$$

Here, $u(x_i)$ are the observed data, $\mathcal{N}[\cdot]$ denotes a differential operator imposing the underlying physics (e.g., from a PDE), N and M are the numbers of data and collocation points, respectively, and λ is a hyperparameter that balances data fidelity and physics enforcement.

This expression illustrates how FBPINNs combine traditional Fourier-based representations with the flexibility of neural networks to learn solutions that adhere to both data and physics constraints. By dividing the problem domain into smaller subdomains and using modular network architectures, these models can efficiently tackle multi-scale and heterogeneous issues. The modular approach allows for parallel training and smooth transitions across subdomain interfaces, enhancing computational efficiency and model robustness. These advancements demonstrate how PINNs have evolved through strategic hybridization, theory-driven constraints, optimized loss formulations, and domain decomposition. Consequently, PINNs have broadened their applicability in various scientific and engineering fields, offering faster and more precise solutions to real-world problems.

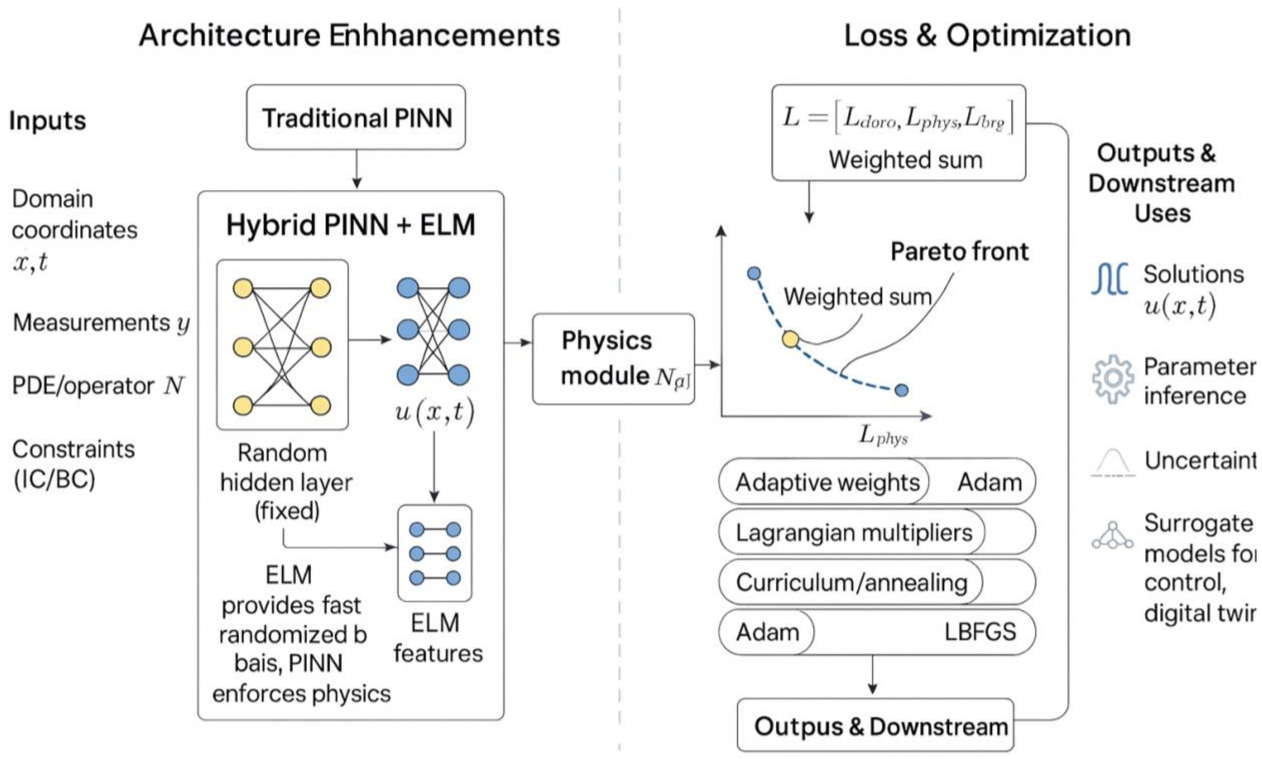

Figure 1: Enhanced PINN architectures with elms and multi-objective loss optimization

1.2. Domain Decomposition and Modular Approaches

Domain decomposition and modular approaches in PIML have evolved significantly with recent innovations such as disjointed PINNs and extended PINNs (XPINNs), which partition complex, multi-scale problems into smaller subdomains to accelerate training and inference while enhancing scalability in the presence of sharp gradients or heterogeneous materials. In this framework, each subdomain is addressed by an individual neural network with optimally selected hyperparameters, thereby facilitating parallel computation and reducing training costs (Jagtap & Karniadakis, 2020). By enabling arbitrary partitioning in both space and time, XPINNs overcome the limitations of traditional PINNs and even the more conservative PINN (cPINN) approaches, as they allow for flexible representation of localized phenomena and improved handling of discontinuities in material properties (Gu et al., 2024). The modular nature of this method permits the integration of diverse multi-physics data, and the disjointed structure supports robust interface coupling through continuity conditions, ultimately leading to enhanced predictive accuracy and convergence (Wu et al., 2024; Shukla et al., 2022). Consequently, these advances not only streamline the computational process but also provide a scalable solution for solving nonlinear partial differential equations in complex domains, marking a significant step forward in the application of physics-informed neural networks for practical engineering challenges.

1.3. Operator Learning and Neural Operators

Physics-informed neural operator (PINO) frameworks represent a major leap forward in PIML. PINOs learn mappings between entire function spaces rather than individual pointwise solutions,

thereby offering discretization invariance – once trained, these models are not tied to a specific grid or mesh and can be evaluated across various discretizations without retraining (Rosofsky et al., 2023). This flexibility is particularly advantageous in applications such as fluid dynamics and weather forecasting, where simulation grids must adapt to different resolution requirements (Nyangon, 2025b). Moreover, PINOs facilitate rapid surrogate modelling by approximating the entire solution operator, which dramatically reduces the computational time compared to traditional numerical methods. In this context, the Physical Invariant Attention Neural Operator (PIANO) has emerged as a novel variant that integrates physical invariants into the model architecture, thereby enhancing performance by effectively deciphering underlying physical laws (Zhang et al., 2025; Li et al., 2024). By embedding discretization invariance and fast surrogate modelling within their framework, these neural operators provide robust and efficient tools for solving complex, multi-scale physical problems.

Enhanced generalization across parameter spaces and scalability to multi-physics problems further underscore the transformative potential of neural operator frameworks. By capturing the intrinsic mapping between functions, PINOs generalize well to new, unseen scenarios, allowing for interpolation and even extrapolation across varying physical conditions without extensive retraining. This robustness is essential for applications involving high-dimensional parameter spaces, where traditional methods falter. Additionally, the ability to integrate physics-based constraints, such as conservation laws, enables these operators to scale efficiently to complex multi-physics problems, including advanced aerospace simulations and climate modelling. The incorporation of techniques like the fast Fourier transform (FFT) in architectures such as the Fourier Neural Operator (FNO) enhances computational efficiency and prediction accuracy (Rosofsky et al., 2023). Collectively, these innovations demonstrate that both PINOs and PIANO offer promising pathways for accelerating simulation processes while maintaining high fidelity in modelling complex, real-world phenomena.

1.4 Neural Networks for the Design and Optimization of Electrical Machines

Adaptive optimization and multi-fidelity frameworks significantly enhance PIML by addressing critical challenges in training PINNs. A key improvement is dynamic loss balancing through adaptive weight tuning, which optimally adjusts the contributions of data loss and physics-based residual loss. By employing multi-objective optimization techniques such as gradient normalization, training processes are stabilized, preventing one loss component from dominating, particularly when data is sparse or noisy (Rohrhofer et al., 2023). Transfer learning techniques further improve convergence efficiency by leveraging pre-trained models, allowing PINNs to optimize across different physical systems. This method reduces sample complexity by fine-tuning networks with low-fidelity or sparse datasets based on high-fidelity simulations, thus accelerating training while preserving predictive accuracy (Xu et al., 2023). Moreover, robust optimization algorithms incorporating second-order techniques, adaptive learning rate schedules, and meta-learning approaches mitigate non-convexity issues in loss landscapes, ensuring stable convergence even in high-dimensional problems (Escapil-Inchauspé & Ruz, 2023).

Multi-fidelity data integration plays a crucial role in improving PIML model accuracy and robustness. By seamlessly combining heterogeneous data sources, such as high-fidelity experimental results with lower-fidelity approximations, predictive performance is enhanced while

computational costs are minimized (Liu & Wang, 2019). This approach enables the use of inexpensive, low-fidelity data to guide training while sparse high-fidelity measurements correct errors and quantify uncertainties at multiple levels. Additionally, conservative PINNs (cPINNs) and XPINNs are two basic methods that use the domain decomposition framework, further improving scalability in large-scale problems (Shukla et al., 2022). To formulate a domain decomposition approach for cPINNs applied to electric machine systems that obey conservation laws, assume a global domain Ω is split into N subdomains,$\Omega = \cup_{i=j}^{N} \Omega_i$, and a separate neural network (or a partition of the network) approximates the solution in each subdomain. The overall loss function can then be built by summing contributions from the residuals (i.e., the conservation laws), the enforcement of boundary conditions, and the continuity conditions across interfaces between subdomains.

Assume that the underlying conservation law (for instance, conservation of charge or energy) can be written abstractly as

$$\mathcal{N}[u(\mathrm{x}, t)] = 0, \quad (\mathbf{x}, t) \in \Omega,$$

where $\mathcal{N}$ is a differential operator (which, in an electric machine system, could arise from Maxwell's equations or other energy/charge conservation equations). In each subdomain Ω_i, let

$$u_i(\mathrm{x}, t; \theta_i)$$

be the neural network approximation of the solution. Then the overall loss can be written as

$$\min_{\{\theta_i\}} \mathcal{L}(\{\theta_i\}) = \sum_{i=1}^{N} \left[\mathcal{L}_{\mathrm{res}}^{(i)} + \lambda_{\mathrm{BC}}\, \mathcal{L}_{\mathrm{BC}}^{(i)}\right] + \lambda_{\mathrm{int}} \sum_{(i,j)\in\mathcal{I}} \mathcal{L}_{\mathrm{int}}^{(i,j)},$$

where:

a) Residual loss in each subdomain (conservation law enforcement):

$$\mathcal{L}_{\mathrm{res}}^{(i)} = \frac{1}{N_i} \sum_{k=1}^{N_i} \| \mathcal{N}[u_i(\mathrm{x}_k, t_k; \theta_i)] \|^2,$$

with $(\mathbf{x}_k, t_k)$ denoting collocation points in subdomain Ω_i.

b) Boundary condition loss in each subdomain:

$$\mathcal{L}_{\mathrm{BC}}^{(i)} = \frac{1}{M_i} \sum_{l=1}^{M_i} \| u_i(\mathrm{x}_l, t_l; \theta_i) - g(\mathrm{x}_l, t_l) \|^2,$$

where g is the prescribed boundary condition function at the boundary points $(\mathbf{x}_l, t_l)$.

c) Interface loss (enforcing continuity across subdomains):

$$\mathcal{L}_{\mathrm{int}}^{(i,j)} = \frac{1}{P_{ij}} \sum_{m=1}^{P_{ij}} \| u_i(\mathrm{x}_m, t_m; \theta_i) - u_j(\mathrm{x}_m, t_m; \theta_j) \|^2,$$

with the sum taken over collocation points $(\mathbf{x}_m, t_m)$ along the interface $\Gamma_{ij} = \partial\Omega_i \cap \partial\Omega_j$ between subdomains Ω_i and Ω_j.

Here, λ_{BC} and λ_{int} are penalty weights that balance the contributions from the boundary and interface terms.

Despite these advancements, PINNs remain difficult to train due to sensitivity in loss weight selection. However, research demonstrates that system parameterization significantly impacts loss scaling, requiring adaptive strategies for optimal performance (Rohrhofer et al., 2023). Advanced frameworks incorporating domain adaptation techniques and automatic loss weighting schemes hold the potential to streamline training, making PIML a powerful tool for applications in geophysics, materials modelling, and fluid mechanics (Guo et al., 2024).

1.5. Integration with Functional Interpolation Techniques

PIML integrated with functional interpolation techniques, such as the Theory of Functional Connections (TFC), is advancing computational methods by enforcing boundary and initial conditions analytically. TFC offers closed-form expressions that satisfy constraints exactly, thereby eliminating the need for "soft" imposition via penalty terms (Laghi et al., 2023; Leake & Mortari, 2020). The core idea behind the TFC is to recast a constrained problem into an unconstrained one by "building in" the constraints exactly into the solution form. In TFC, one typically expresses the unknown function as the sum of two parts: (a) a particular "base" function that satisfies the constraints, and (b) a free function (often denoted $g(x)$) multiplied by "blending" functions that vanish at the constraint locations. A general TFC formulation for a problem with linear constraints can be written as:

$$f(x) = g(x) + \sum_{i=1}^{m} \psi_i(x)(c_i - \mathcal{L}_i\{g(x)\}),$$

where:

- $\mathcal{L}_i\{f(x)\} = c_i$ for $i = 1, \ldots, m$ represent the linear constraints (for example, boundary or initial conditions),
- c_i are the prescribed values,
- $g(x)$ is an arbitrary free function, and
- $\psi_i(x)$ are carefully chosen functions that satisfy the conditions

$$\mathcal{L}_j\{\psi_i(x)\} = \delta_{ij}, \quad j, i = 1, \ldots, m,$$

with δ_{ij} being the Kronecker delta. This guarantees that when the operator $\mathcal{L}_j$ is applied to $f(x)$, the corrective terms exactly "cancel out" any deviation from the constraint caused by the free function $g(x)$. This analytical enforcement restricts the neural network to a subspace of functions that inherently meet the problem's constraints, reducing the search space and minimizing the risk of converging to spurious or non-physical solutions. Furthermore, by removing the competing objectives between data fit and constraint satisfaction, the optimization process experiences enhanced convergence rates and improved numerical stability, particularly in stiff or high-

dimensional scenarios (De Florio et al., 2022). Such benefits not only bolster model reliability but also ease the computational burden during training.

Furthermore, integrating TFC with PINNs promotes improved scalability and generalization across complex domains. With constraints handled analytically, models can generalize more effectively over varied discretizations and domain geometries, a vital advantage for multi-scale and multi-physics challenges (Koyama et al., 2024). This approach has demonstrated success in fields such as fluid mechanics, chemical engineering, and tribology, where predictive performance and interpretability are crucial (Marian & Tremmel, 2023). Recent frameworks—like the Extreme Theory of Functional Connections (X-TFC)—enhance these capabilities by combining shallow neural networks with random feature techniques, ensuring robust solutions even with limited or perturbed data (Schiassi et al., 2021). Collectively, these developments signify a shift towards more accurate, interpretable, and computationally efficient models in solving differential equations.

2. Applications of PIML in Electrical Machines and Drives

2.1 Field Analysis and Simulation

In electrical machines and drives, physics consistency is fundamental to ensuring that electromagnetic field simulations accurately reflect real-world behavior. Central to this consistency are Maxwell's equations—Gauss's law for electricity, Gauss's law for magnetism, Faraday's law of induction, and Ampère's law with Maxwell's correction—which together describe how electric and magnetic fields are generated and interact with charges and currents (Watthewaduge et al., 2020). To compute the electromagnetic torque in an electrical machine, one typically begins with the full set of Maxwell's equations and then uses them to derive the forces and moments acting on the machine's moving parts. The key steps involve:

(a) Maxwell's equations and constitutive relations:

The differential form of Maxwell's equations are given by:

$$\nabla \cdot \mathbf{D} = \rho \quad \text{(Gauss's law for electricity)}$$

$$\nabla \cdot \mathbf{B} = 0 \quad \text{(Gauss's law for magnetism)}$$

$$\nabla \times \mathbf{E} = -\frac{\partial \mathbf{B}}{\partial t} \quad \text{(Faraday's law)}$$

$$\nabla \times \mathbf{H} = \mathbf{J} + \frac{\partial \mathbf{D}}{\partial t} \quad \text{(Ampere's law with Maxwell's correction)}$$

The fields are linked by the constitutive relations, which in a linear medium are:

$$\mathbf{D} = \varepsilon \mathbf{E}, \quad \mathbf{B} = \mu \mathbf{H},$$

where,

- ε is the permittivity,
- μ is the permeability,
- ρ is the charge density, and
- J is the current density.

(b) Force and torque density:

The Lorentz force density, representing the force per unit volume, is expressed as:

$$\mathrm{f} = \rho\,\mathbf{E} + \mathbf{J} \times \mathbf{B}.$$

The electromagnetic torque T acting on a volume V can then be calculated by taking the moment of the force density:

$$\mathbf{T} = \int_{\mathrm{V}} \mathbf{r} \times \mathbf{f}\,\mathrm{dV},$$

where r is the position vector relative to the axis of rotation.

(c) Maxwell stress tensor approach:

A more general and often more convenient formulation is to express the torque using the Maxwell stress tensor. The Maxwell stress tensor T_{stress} for a medium with permittivity ε and permeability μ is defined as:

$$\mathbf{T}_{\mathrm{stress}} = \mathbf{DE} + \mathbf{BH} - \frac{1}{2}(\mathbf{E} \cdot \mathbf{D} + \mathrm{B} \cdot \mathbf{H})\mathrm{I},$$

where I is the identity tensor. Here, the dyadic products DE and BH represent second-order tensors formed from the field vectors.

The torque can then be computed by integrating the moment due to the stress tensor over a closed surface S that encloses the volume of interest:

$$\mathbf{T} = \oint_{\mathrm{S}} \mathbf{r} \times (\mathbf{T}_{\mathrm{stress}} \cdot \mathbf{n})\mathrm{dS},$$

where n is the outward unit normal to the surface S.

(d) Application in electrical machines: In electrical machines, such as motors or drives, the interaction between the stator and rotor plays a critical role in their operation.

- Stator and rotor interaction: The stator windings generate a time-varying magnetic field, as described by Ampère's law. This field then interacts with the currents induced in the rotor, or with pre-existing rotor currents in the case of synchronous machines, generating a Lorentz force.
- Torque production: The distribution of electromagnetic fields, which is determined by Maxwell's equations, dictates the force density within the machine. By integrating the moments of these forces (or using the Maxwell stress tensor method) over the geometry of the machine, the net electromagnetic torque that drives the rotor can be computed.

This formulation links the fundamental Maxwell's equations directly to the torque computation, facilitating the accurate modelling and simulation of complex electromagnetic devices. Combining

these elements, a detailed expression for the electromagnetic torque T in an electrical machine can be written as:

$$T = \oint_S r \times \left\{ \left[DE + BH - \frac{1}{2}(E \cdot D + B \cdot H)I \right] \cdot n \right\} dS.$$

This integral formulation can be used to compute the torque based on the distribution of electric and magnetic fields, which are determined by solving Maxwell's equations with appropriate boundary conditions and material properties within the machine. These equations provide the theoretical foundation for calculating electromagnetic torque in electrical machines and drives. Additionally, they inform the selection of boundary conditions, such as Dirichlet and Neumann types, which govern the field behavior at material interfaces. Advanced numerical methods like the finite element method (FEM) and finite difference method (FDM) are applied to discretize the domain and solve these equations, thereby preserving both local field details and global performance characteristics (Roubache et al., 2018). This rigorous application of theory ensures that the simulation honors fundamental physical laws, enabling accurate predictions across varied operating conditions. In addition, cross-validation against analytical solutions and experimental measurements reinforces the robustness of these models, while continual refinements in solver algorithms further enhance computational reliability.

Data integration in field analysis requires leveraging sparse experimental or sensor data to calibrate and validate simulation models effectively. Techniques such as data assimilation and transfer learning enable the integration of limited sensor readings with high-fidelity numerical simulations, thus bridging the gap between theoretical predictions and practical measurements (Alatawneh & Pillay, 2016). By incorporating sparse data into the calibration process, researchers can refine simulation outputs and adjust model parameters, which in turn improves the overall accuracy of field predictions. Simultaneously, accounting for material nonlinearity—such as magnetic saturation and hysteresis effects—is crucial for capturing the true behavior of electrical machines. Advanced control strategies and iterative methods, including feedback linearization and refined current sheet models, have been developed to address these nonlinearities (Accetta et al., 2022; J. Guo et al., 2020). These approaches not only capture the inherent nonlinearity of magnetic materials but also mitigate instability in simulations. Furthermore, by integrating sensor calibration results with nonlinear solvers, engineers can enhance model fidelity, ensuring that both local material behaviors and broader field interactions are accurately represented. This synthesis of sparse data and nonlinear modelling is vital for developing reliable simulations that can support advanced machine design and operational optimization.

Achieving computational efficiency without sacrificing simulation fidelity is a constant challenge in field analysis for electrical machines and drives. High-fidelity simulations, while essential for capturing intricate electromagnetic interactions, must be balanced with real-time or iterative design requirements. Surrogate modelling techniques, mesh optimization strategies, and hardware acceleration (via FPGAs or GPUs) have emerged as effective solutions to reduce computational overhead (Mojlish et al., 2017; Praslicka et al., 2023; Tahkola et al., 2020). These methods optimize mesh resolution and solver performance, allowing detailed simulations to be performed more rapidly. Equally important is the incorporation of error bounds and probabilistic measures to

assess the reliability of simulation predictions. Techniques such as Bayesian calibration, Monte Carlo methods, and Gaussian process regression provide quantitative estimates of uncertainty, enabling engineers to gauge the confidence of their results (Ma et al., 2022; Manfredi & Trinchero, 2022). By combining high-fidelity physics-based models with data-driven uncertainty quantification frameworks, researchers can deliver robust field predictions that inform both design optimization and risk assessment. Ultimately, this integrated approach supports the development of electrical machines and drives that are not only high performing but also resilient to variabilities in material properties and operational conditions.

2. Benchmarking PIML Architectures: Scalability, Data Efficiency, Robustness, and Real-Time Deployability

Below are four structured comparison tables synthesizing the key dimensions discussed across the foregoing sections. Each table compares the major PIML approaches—PINNs, PIGNNs, Neural Operators (DeepONets/FNOs), and Physics-Informed Gaussian Processes (PIGPs)—against Traditional ML and Classical Numerical Methods (FEM/FDM) as baselines.

Table 1: Scalability

**Approach**	**Geometric Scalability**	**Dimensional Scalability**	**Industrial Deployment Readiness**	**Key Evidence / Limitation**
Classical FEM/FDM	High (mature meshing)	Poor at high dimensions	Established but slow	3–5 hr simulations per sample; computationally prohibitive for real-time use (Parekh et al., 2023)
Traditional ML	Limited; geometry-agnostic but data-hungry	Moderate	High once trained, but brittle	No physical scaling guarantees; degrades on unseen topologies (Wu et al., 2024)
PINNs	Moderate; mesh-free advantage offset by per-geometry retraining	Poor for stiff/multi-scale regimes	Low–Moderate	Convergence degrades sharply with domain complexity (Karniadakis et al., 2021)
PIGNNs	High; native unstructured-mesh handling	Moderate	Moderate	Over-smoothing as message-passing depth increases (Zhao et al., 2022)
Neural Operators (DeepONets/FNOs)	FNO: regular grids only; DeepONet: mesh-agnostic	High; learn across function families	Moderate–High	Single trained model generalizes across BCs/excitations (Sharma et al., 2023)

PIGPs	Low–Moderate; cubic scaling in data points	Moderate	Low for large datasets	Best suited to sparse-data regimes (Huber et al., 2023)

Table 1: Data Efficiency

**Approach**	**Sparse-Data Performance**	**Sensitivity to Noise**	**Reliance on Labelled Data**	**Key Evidence**
Classical FEM/FDM	N/A (no training)	Low (deterministic)	None	Requires accurate material/boundary specs
Traditional ML	Poor	High	Heavy	Performance collapses under data scarcity (Gawande, 2024)
PINNs	Moderate; physics acts as regularizer	Moderate–High; unstable error propagation	Light	Vanilla PINNs propagate boundary noise unstably (Bajaj et al., 2023)
PIGNNs	Moderate–High	Moderate	Moderate	Topological priors aid generalization (Zhao et al., 2022)
Neural Operators	Moderate	Moderate	Heavy (paired functions)	DeepONets need substantial training pairs (Sharma et al., 2023)
PIGPs	High	Low (probabilistic averaging)	Light	Native uncertainty quantification under sparsity (Huber et al., 2023)

Table 3: Robustness

**Approach**	**Handles Nonlinearity (Saturation/Hysteresis)**	**Uncertainty Quantification**	**Generalization Across Operating Conditions**	**Known Failure Modes**
Classical FEM/FDM	Yes, with nonlinear solvers	None native	Limited; case-by-case	Computational blow-up under stiffness
Traditional ML	Limited	Limited (ensembles only)	Poor outside training distribution	No physical consistency (Wu et al., 2024)
PINNs	Moderate	Limited	Poor across geometries	Spectral bias; gradient imbalance among loss terms (Krishnapriyan et al., 2021)

PIGNNs	Moderate–High	Limited	Moderate	Long-range field interactions poorly propagated (Zhao et al., 2022)
Neural Operators	High	Limited (deterministic)	High across BCs/excitations	FNO struggles with sharp iron–air discontinuities (Sharma et al., 2023)
PIGPs	Moderate	Native (Bayesian)	Moderate	Scalability ceiling on large datasets (Huber et al., 2023)
Hybrid (NO + PINN residual; PIGNN + PIGP)	High	High	High	Architectural complexity; tuning burden (Zideh et al., 2024)

Table 3: Real-Time Deployability

**Approach**	**Inference Latency**	**Suitability for Digital Twins**	**Hardware Acceleration Compatibility**	**Industry 4.0 Integration**
Classical FEM/FDM	Seconds–hours per sample	Offline calibration only	Limited	Poor for real-time control (Parekh et al., 2023)
Traditional ML	Sub-millisecond	High once trained	Excellent (GPU)	Already deployed; lacks physics fidelity
PINNs	Milliseconds (post-training)	Moderate	Good (GPU/automatic diff)	Limited by per-case retraining (Bajaj et al., 2023)
PIGNNs	Milliseconds	High; aligns with FE meshes	Good	Promising for fault diagnostics (Zhao et al., 2022)
Neural Operators	~100 ms per sample(vs. 3–5 hr FEM)	High	Excellent	Strong fit for design exploration & MPC (Parekh et al., 2023)
PIGPs	Moderate (depends on data size)	Moderate	Limited	Best for low-throughput, high-stakes decisions (Huber et al., 2023)

PIBO-MESA (optimization layer)	~45% faster than NSGA-II	High	Good	Validated in design-loop deployment (Asef & Vagg, 2024)

Across all four dimensions, no single PIML approach dominates. PINNs offer mesh-free flexibility but suffer spectral bias and per-geometry retraining; PIGNNs excel on unstructured electromagnetic meshes but face over-smoothing; neural operators deliver the strongest real-time and cross-condition generalization but are data-hungry and grid-constrained (FNOs) or paired-data-dependent (DeepONets); PIGPs uniquely deliver native uncertainty quantification under sparsity but scale poorly. Hybrid architectures—combining neural operators for global field prediction with PINN residuals for local enforcement, or coupling PIGNNs with PIGPs for uncertainty-aware diagnostics - are emerging as the most pragmatic path to industrial-grade deployment under Industry 4.0 (Cheruku et al., 2025; Zideh et al., 2024).

References

Accetta, A., Cirrincione, M., Pucci, M., & Sferlazza, A. (2022). Feedback Linearization Based Nonlinear Control of SynRM Drives Accounting for Self- and Cross-Saturation. *IEEE Transactions on Industry Applications*, *58*(3), 3637–3651. IEEE Transactions on Industry Applications. https://doi.org/10.1109/TIA.2022.3155511

Alatawneh, N., & Pillay, P. (2016). Calibration of the Tangential Coil Sensor for the Measurement of Core Losses in Electrical Machine Laminations. *IEEE Transactions on Energy Conversion*, *31*(2), 413–423. IEEE Transactions on Energy Conversion. https://doi.org/10.1109/TEC.2016.2525011

De Florio, M., Schiassi, E., & Furfaro, R. (2022). Physics-informed neural networks and functional interpolation for stiff chemical kinetics. *Chaos: An Interdisciplinary Journal of Nonlinear Science*, *32*(6), 063107. https://doi.org/10.1063/5.0086649

Escapil-Inchauspé, P., & Ruz, G. A. (2023). H-Analysis and data-parallel physics-informed neural networks. *Scientific Reports*, *13*(1), 17562. https://doi.org/10.1038/s41598-023-44541-5

Gu, L., Qin, S., Xu, L., & Chen, R. (2024). Physics-informed neural networks with domain decomposition for the incompressible Navier–Stokes equations. *Physics of Fluids*, *36*(2), 021914. https://doi.org/10.1063/5.0188830

Guo, J., Quéval, L., Roucaries, B., Vido, L., Liu, L., Trillaud, F., & Berriaud, C. (2020). Nonlinear Current Sheet Model of Electrical Machines. *IEEE Transactions on Magnetics*, *56*(1), 1–4. IEEE Transactions on Magnetics. https://doi.org/10.1109/TMAG.2019.2950614

Guo, X., Hu, X., & Zhang, S. (2024). Application status of variable-frequency drive in hydrogen fuel cell air compressors from an industrial viewpoint: A review. *Sustainable Energy Technologies and Assessments*, *64*, 103716. https://doi.org/10.1016/j.seta.2024.103716

Jagtap, A. D., & Karniadakis, G. E. (2020). Extended Physics-Informed Neural Networks (XPINNs): A Generalized Space-Time Domain Decomposition Based Deep Learning Framework for Nonlinear Partial Differential Equations. *Communications in Computational Physics*, *31*(5), 2002–2041. https://doi.org/10.4208/cicp.OA-2020-0164

Joshi, K., Snigdha, V., & Bhattacharya, A. K. (2024). Physics Informed Extreme Learning Machines With Residual Variation Diminishing Scheme for Nonlinear Problems With Discontinuous Surfaces. *IEEE Access*, *12*, 130617–130629. IEEE Access. https://doi.org/10.1109/ACCESS.2024.3457670

Khademi, A., & Dufour, S. (2024). A novel discretized physics-informed neural network model applied to the Navier–Stokes equations. *Physica Scripta*, *99*(7), 076016. https://doi.org/10.1088/1402-4896/ad5592

Koyama, S., Ribeiro, J. G. C., Nakamura, T., Ueno, N., & Pezzoli, M. (2024). Physics-Informed Machine Learning for Sound Field Estimation: Fundamentals, state of the art, and challenges. *IEEE Signal Processing Magazine*, *41*(6), 60–71. IEEE Signal Processing Magazine. https://doi.org/10.1109/MSP.2024.3465896

Laghi, L., Schiassi, E., De Florio, M., Furfaro, R., & Mostacci, D. (2023). Physics-Informed Neural Networks for 1-D Steady-State Diffusion-Advection-Reaction Equations. *Nuclear Science and Engineering*, *197*(9), 2373–2403. https://doi.org/10.1080/00295639.2022.2160604

Leake, C., & Mortari, D. (2020). Deep Theory of Functional Connections: A New Method for Estimating the Solutions of Partial Differential Equations. *Machine Learning and Knowledge Extraction*, *2*(1), Article 1. https://doi.org/10.3390/make2010004

Li, S., Liu, C., & Ni, H. (2024). Enhancing neural operator learning with invariants to simultaneously learn various physical mechanisms. *National Science Review*, *11*(8), nwae198. https://doi.org/10.1093/nsr/nwae198

Liu, D., & Wang, Y. (2019). Multi-Fidelity Physics-Constrained Neural Network and Its Application in Materials Modeling. *Journal of Mechanical Design*, *141*(121403). https://doi.org/10.1115/1.4044400

Ma, X., Zhang, Z., & Hua, H. (2022). Uncertainty quantization and reliability analysis for rotor/stator rub-impact using advanced Kriging surrogate model. *Journal of Sound and Vibration*, *525*, 116800. https://doi.org/10.1016/j.jsv.2022.116800

Manfredi, P., & Trinchero, R. (2022). A Probabilistic Machine Learning Approach for the Uncertainty Quantification of Electronic Circuits Based on Gaussian Process Regression. *IEEE Transactions on Computer-Aided Design of Integrated Circuits and Systems*, *41*(8), 2638–2651. IEEE Transactions on Computer-Aided Design of Integrated Circuits and Systems. https://doi.org/10.1109/TCAD.2021.3112138

Marian, M., & Tremmel, S. (2023). Physics-Informed Machine Learning—An Emerging Trend in Tribology. *Lubricants*, *11*(11), Article 11. https://doi.org/10.3390/lubricants11110463

Mojlish, S., Erdogan, N., Levine, D., & Davoudi, A. (2017). Review of Hardware Platforms for Real-Time Simulation of Electric Machines. *IEEE Transactions on Transportation Electrification*, *3*(1), 130–146. IEEE Transactions on Transportation Electrification. https://doi.org/10.1109/TTE.2017.2656141

Moseley, B., Markham, A., & Nissen-Meyer, T. (2023). Finite basis physics-informed neural networks (FBPINNs): A scalable domain decomposition approach for solving differential equations. *Advances in Computational Mathematics*, *49*(4), 62. https://doi.org/10.1007/s10444-023-10065-9

Nyangon, J. (2025a). Physics informed neural networks for maritime energy systems and blue economy innovations. *Machine Learning: Earth*, *1*(1), 011002. https://doi.org/10.1088/3049-4753/adfe73

Nyangon, J. (2025b). Smart Grid Strategies for Tackling the Duck Curve: A Qualitative Assessment of Digitalization, Battery Energy Storage, and Managed Rebound Effects Benefits. *Energies*, *18*(15), 3988. https://doi.org/10.3390/en18153988

Praslicka, B., Ma, C., & Taran, N. (2023). A Computationally Efficient High-Fidelity Multi-Physics Design Optimization of Traction Motors for Drive Cycle Loss Minimization. *IEEE Transactions on Industry Applications*, *59*(2), 1351–1360. IEEE Transactions on Industry Applications. https://doi.org/10.1109/TIA.2022.3220554

Rohrhofer, F. M., Posch, S., Gößnitzer, C., & Geiger, B. C. (2023). Data vs. Physics: The Apparent Pareto Front of Physics-Informed Neural Networks. *IEEE Access*, *11*, 86252–86261. IEEE Access. https://doi.org/10.1109/ACCESS.2023.3302892

Rosofsky, S. G., Al Majed, H., & Huerta, E. A. (2023). Applications of physics informed neural operators. *Machine Learning: Science and Technology*, *4*(2), 025022. https://doi.org/10.1088/2632-2153/acd168

Roubache, L., Boughrara, K., Dubas, F., & Ibtiouen, R. (2018). New Subdomain Technique for Electromagnetic Performances Calculation in Radial-Flux Electrical Machines Considering Finite Soft-Magnetic Material Permeability. *IEEE Transactions on Magnetics*, *54*(4), 1–15. IEEE Transactions on Magnetics. https://doi.org/10.1109/TMAG.2017.2785254

Schiassi, E., De Florio, M., D'Ambrosio, A., Mortari, D., & Furfaro, R. (2021). Physics-Informed Neural Networks and Functional Interpolation for Data-Driven Parameters Discovery of

Epidemiological Compartmental Models. *Mathematics*, *9*(17), Article 17. https://doi.org/10.3390/math9172069
Shukla, K., Xu, M., Trask, N., & Karniadakis, G. E. (2022). Scalable algorithms for physics-informed neural and graph networks. *Data-Centric Engineering*, *3*, e24. https://doi.org/10.1017/dce.2022.24
Tahkola, M., Keränen, J., Sedov, D., Far, M. F., & Kortelainen, J. (2020). Surrogate Modeling of Electrical Machine Torque Using Artificial Neural Networks. *IEEE Access*, *8*, 220027–220045. IEEE Access. https://doi.org/10.1109/ACCESS.2020.3042834
Watthewaduge, G., Sayed, E., Emadi, A., & Bilgin, B. (2020). Electromagnetic Modeling Techniques for Switched Reluctance Machines: State-of-the-Art Review. *IEEE Open Journal of the Industrial Electronics Society*, *1*, 218–234. IEEE Open Journal of the Industrial Electronics Society. https://doi.org/10.1109/OJIES.2020.3016242
Wu, Y., Sicard, B., & Gadsden, S. A. (2024). Physics-informed machine learning: A comprehensive review on applications in anomaly detection and condition monitoring. *Expert Systems with Applications*, *255*, 124678. https://doi.org/10.1016/j.eswa.2024.124678
Xu, Y., Kohtz, S., Boakye, J., Gardoni, P., & Wang, P. (2023). Physics-informed machine learning for reliability and systems safety applications: State of the art and challenges. *Reliability Engineering & System Safety*, *230*, 108900. https://doi.org/10.1016/j.ress.2022.108900
Zhang, H., Jiang, L., Chu, X., Wen, Y., Li, L., Liu, J., Xiao, Y., & Wang, L. (2025). Combining physics-informed graph neural network and finite difference for solving forward and inverse spatiotemporal PDEs. *Computer Physics Communications*, *308*, 109462. https://doi.org/10.1016/j.cpc.2024.109462
Zhang, S., Wallscheid, O., & Porrmann, M. (2023). Machine Learning for the Control and Monitoring of Electric Machine Drives: Advances and Trends. *IEEE Open Journal of Industry Applications*, *4*, 188–214. IEEE Open Journal of Industry Applications. https://doi.org/10.1109/OJIA.2023.3284717
